# Endogenous feline leukemia virus long terminal repeat integration site diversity is highly variable in related and unrelated domestic cats

**DOI:** 10.1186/s12977-024-00635-0

**Published:** 2024-02-12

**Authors:** Elliott S. Chiu, Coby A. McDonald, Roderick B. Gagne, Henry Dunkleberger, Matthew Moxcey, Sue VandeWoude

**Affiliations:** 1 Department of Microbiology, Immunology, and Pathology, Colorado State University, Fort Collins, CO 80523 USA; 2 Independent Contractor, Fort Collins, CO 80524 USA

**Keywords:** Endogenous retrovirus, Functional genomics, Feline leukemia virus, Integration sites

## Abstract

Endogenous retroviruses (ERV) are indicators of vertebrate evolutionary history and play important roles as homeostatic regulators. ERV long terminal repeat (LTR) elements may act as cis-activating promoters or trans-activating enhancer elements modifying gene transcription distant from LTR insertion sites. We previously documented that endogenous feline leukemia virus (FeLV)-LTR copy number variation in individual cats tracks inversely with susceptibility to virulent FeLV disease. To evaluate FeLV-LTR insertion characteristics, we assessed enFeLV-LTR integration site diversity in 20 cats from three genetically distinct populations using a baited linker-mediated PCR approach. We documented 765 individual integration sites unequally represented among individuals. Only three LTR integration sites were shared among all individuals, while 412 sites were unique to a single individual. When primary fibroblast cultures were challenged with exogenous FeLV, we found significantly increased expression of both exogenous and endogenous FeLV orthologs, supporting previous findings of potential exFeLV-enFeLV interactions; however, viral challenge did not elicit transcriptional changes in genes associated with the vast majority of integration sites. This study assesses FeLV-LTR integration sites in individual animals, providing unique transposome genotypes. Further, we document substantial individual variation in LTR integration site locations, even in a highly inbred population, and provide a framework for understanding potential endogenous retroviral element position influence on host gene transcription.

**Supplementary Information:**

The online version contains supplementary material available at 10.1186/s12977-024-00635-0.

## Introduction

Endogenous retroviruses (ERV) are remnants of ancient viral infections integrated into the genome. ERVs no longer replicate, but may interact with related, circulating exogenous retroviruses [5, 10]. All animals harbor some form of retroviral DNA; human genomes contain 8% DNA that is considered of viral (namely retroviral) origin [13]. Through the course of ERV-host evolution, these viral remnants have assumed many normal cellular processes, including immune modulation, placentation, and oncogenesis [2, 14, 17, 20]. Common interactions between ERVs and their exogenous retrovirus (XRV) counterparts (summarized in [10] are recombination, where the genotypes of two related viruses are combined due to template switching during reverse transcription [16, 26], and receptor interference, where envelope proteins (Env) that are responsible for cellular entry bind and inactivate receptors intracellularly before they can be transported to the cell surface [35]. Less commonly, clonal deletion of T-cells [21] and dysregulation of virion trafficking [32] can result from specific proteins that are similar between ERV-XRV dyads.

Exogenous retroviral genomes contain at minimum three fundamental genes—*gag*, *pol*, and *env*—flanked on both ends by long terminal repeats (LTRs) oriented in the same direction [12]. LTRs contain three regions, the U5, R, and U3 regions, containing enhancer, promoter, and regulatory functions [12]. Genomic LTR copy number is not directly associated with full-length ERV copy number, as ERVs undergo retrotransposition and homologous recombination, resulting in replication, insertion, and deletion of LTR elements at varying sites across the genome. During homologous recombination, LTRs can become disarticulated from their ERV progenitors [43]. In some cases, LTRs maintain their enhancer and promoter functions, which serve important biological purposes, including the regulation of normal gene functions [39], though evidence supports that not all LTRs are functional [18].

In theory, ERV-derived solo-LTRs may indirectly augment host gene expression via two primary mechanisms: *cis*-activation of host genes via the promoter, and *trans*-activation of host genes via the enhancer [39]. Evidently, both these interactions occur with endogenous retroelements, although enhancer functions are more difficult to detect and attribute to specific LTR loci [39] since enhancers modulate transcription of host genes up to 1Mbp away [7, 28, 33]. While not all solo-LTRs will modulate transcription of host genes, those that do likely have selective advantages that result in retention of the LTR in the host genome. For example, the expression of the anti-viral APOBEC gene is promoted by a specific xenotropic murine leukemia virus (MuLV)-derived LTR locus [38].

Feline leukemia virus (FeLV) is a gammaretrovirus that circulates in felids, though the primary host is the domestic cat. Full-length enFeLV proviral copy numbers vary by approximately 8–12 copies per cell [8], while LTR copy number may exceed 80 copies per cell [11, 34]. Horizontally-transmitted exogenous FeLV circulates globally with a variety of outcomes in felid hosts [40]. We have documented a negative dose-dependent relationship between enFeLV-LTR copy number and viral antigen production and exogenous viral load following natural infections [11, 34]. While the production of small RNAs targeting FeLV is correlated with restriction of FeLV antigen production (considered a proxy for replication) [9], we reasoned that restriction of exogenous infection properties of enFeLV-LTRs may also be associated with enhancer and promoter functions that are loci-dependent [4]. Therefore, we examined endogenous feline leukemia virus (enFeLV) LTR integration site variation and associated gene expression in three populations of cats. We characterized fixed, conserved, and unique LTR insertion sites to define an ERV-specific genomic map for each individual, and scrutinized host genes closely associated with conserved integration sites. We identified genes that may indirectly result in exFeLV disease modulation by acting as a restriction factor or activator of immune responses that diminish exFeLV infection, and document increased exogenous and endogenous gene expression during infections. More importantly, we identify unique enFeLV-LTR insertion site maps (i.e., enFeLV-transposomes) of each individual that may relate to phenotypic differences between otherwise highly related individuals.

## Materials and methods

### Sample collection, and DNA & RNA extraction

Blood was collected from seven specific pathogen free (SPF) domestic cats (population 1), and six cats from a privately held domestic cat colony that had been crossed with Asian leopard cats (which lacks enFeLV) more than 5 generations previously (*Prionailurus bengalensis*; population 2, previously described in [34]. Known matrilinear relationships between individuals in population 1 are summarized in Additional file 1: Figure S1. Only one individual (4460) does not share known direct grand-parentage. Full thickness skin biopsies were collected from seven outbred domestic cats during necropsies performed at the Colorado State University Veterinary Diagnostic Laboratory (population 3). Primary fibroblasts were isolated from plated minced skin [42], cultured, and expanded in 20% FBS-supplemented DMEM high glucose media and 1 × antibiotic–antimycotic solution (Gibco, penicillin/streptomycin/fungizone). Peripheral blood mononuclear cells (PBMCs) were isolated from fresh blood by ficoll-gradient centrifugation as previously described [36]. PBMCs were cultured in 20% FBS-supplemented RPMI media supplemented with 100 ng/mL interleukin-2 (Sigma, USA) and 50 ng/mL concanavalin A (Sigma, USA). Blood samples from population 2 were collected, processed, and viably frozen using previously reported methods [34]. DNA was extracted from PBMCs (population 1, SPF and population 2, hybrid) and fibroblasts (population 3, outbred) using a DNeasy blood and tissue DNA extraction kit (Qiagen, US). Endogenous FeLV copy number was quantified in a subset of cats using methods previously reported in [11].

### RNA preparation from FeLV-infected fibroblasts and PBMCs

A subset of fibroblasts were infected with an MOI of 0.01 subgroup A FeLV-61E [22] and cultured for 5 days prior to total cellular RNA extraction. RNA was extracted from six of the seven uninfected PBMCs from population 1 and five of the six uninfected and FeLV-infected fibroblasts from population 3 using an RNAeasy kit (Qiagen, US).

### DNA Linker-mediated PCR, RNA library prep, and sequencing

**Fig. 1:**
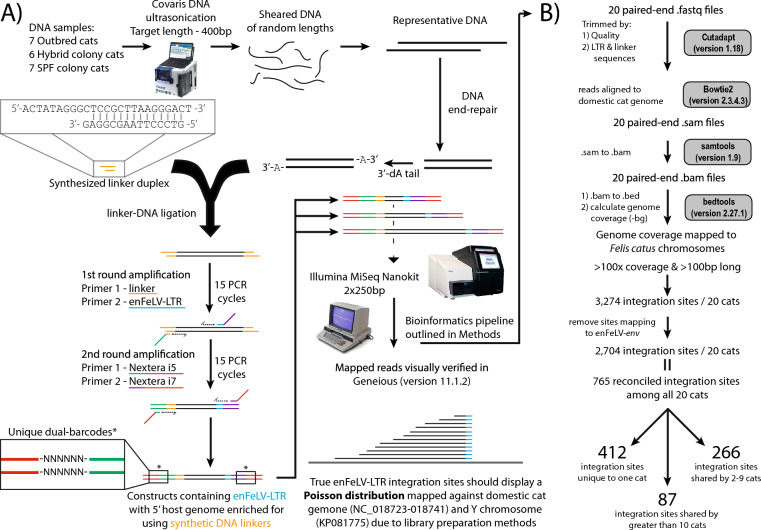
Illustrated library preparation and bioinformatics pipeline. **A** DNA from outbred (n = 7), hybrid colony (n = 6), and SPF colony (n = 7) domestic cats was sheared and ligated to synthesized linkers to selectively amplify enFeLV-LTR integration sites. Due to library preparation methods, integration sites displayed a Poisson distribution. **B** Bioinfomatics pipeline used to generate mapped LTR integration identified unique and common integration sites shared between all cats included in this study

In order to identify FeLV-LTR integration sites, we employed a strategy to directly enrich sequencing results for LTR integrations, adapted from [27] (Fig. 1a. DNA was sheared by ultrasonication using a Covaris M220, with specifications to target DNA fragments with an average length of 400 base pairs (Peak incident power, 50; 200 cycles per burst; duty factor, 10%; 70 s. Sheared DNA was further size-selected with AMPure beads (Agilent at a concentration of 0.8 × beads. Enzyme-facilitated end repair and 3ʹ-dA-tailing was completed under manufacturer’s directions (New England Biolabs, Ipswich, MA. Duplexed linker sequences 5ʹ-ACTATAGGGCTCCGCTTAAGGGACT-3ʹ and 5ʹ-GTCCCTTAAGCGGAG-3ʹ were synthesized with a 3ʹ-T overhang. Linkers (500 nM) were ligated to size-selected, sheared DNA with T4 DNA Ligase (New England BioLabs). Flanking 3ʹ-dT’s on the linker and 3’-dA’s on the DNA selected against the formation of DNA-DNA and linker-linker complexes. Unligated linkers were removed with an AMPure bead cleanup at a concentration of 0.8 × beads. LTR-integration sites were enriched using a PCR with Illumina adapter sequences adjoined to either enFeLV-LTR-specific sequence or the synthetic linker sequence (5ʹ-GTCTCGTGGGCTCGGAGATGTGTATAAGAGACAG*TAAACGGTGGACTTAGGATAAGAC* -3ʹ [enFeLV-LTR-specific sequence italicized] and TCGTCGGCAGCGTCAGATGTGTATAAGAGACAG*ACTATAGGGCTCCGCTTAA* -3ʹ [synthetic linker sequence italicized]). The LTR-specific sequence is highly conserved reported by publicly available enFeLV and exogenous FeLV sequences. Second round primers contained unique barcodes to allow for identification of individual samples. Each round was 15 cycles and completed using 9 µl of template, 10 µl of KAPA high fidelity polymerase, and 1 µl of primer and an annealing temp of 60 ℃. Libraries were purified and size selected with AMPure beads at a concentration of 0.6 × beads. Quality control of the library was performed after each step of library preparation using a High Sensitivity D1000 DNA tape on an Agilent 2200 tape station (Agilent, US). The 20 unique dual-indexed fibroblast and PBMC libraries (Accession numbers: SRR23085866-SRR23085878) were combined and sequenced on an Illumina MiSeq using a MiSeq Reagent 2 × 250bp Nano kit v2 (Illumina, San Diego, CA) at Colorado State University’s Next Generation Sequencing Core.

We performed RNA-sequencing on a subset of cats from populations 1 and 3. RNA was extracted from PBMCs from six cats from population 1 and from infected and non-infected fibroblasts from five cats from population 3. Extracted RNA was sent to the University of Colorado, Anschutz genomics and microarray core for library preparation with ribosomal RNA depletion as well as total transcriptome sequencing. Libraries were run on one lane of an Illumina NovaSeq 6000 and sequenced 2 × 150bp (Accession numbers: SRR23085859-SRR23085865).

### Bioinformatics analysis: integration sites

Domestic cat LTR integration sites were determined using a custom bioinformatics pipeline (Fig. 1B; https://github.com/VandeWoude-Laboratory). Briefly, reads were filtered for quality and adapters trimmed using cutadapt (version 2.7). Reads were aligned with Bowtie2 (version 2.3.4.3) with a minimum local alignment score of 50 to the *Felis catus* whole genome 9.0 (NC_018723-NC_018741) with the addition of the domestic cat Y-chromosome (KP081775). The resulting.sam files were converted to.bam files using samtools (version 1.9). Bedtools (version 2.27.1) genomecov –bg function was used to calculate genome coverage in both exonic and intronic regions with collapsed viewing format. LTR integration sites were filtered based on length of integration site (> 100 bp; < 1000 bp) and minimum sequence depth (> 100x). The most recent domestic cat genome (F.catus_Fca126_mat1.0) was not available at the time of this work.

LTR integration sites were confirmed visually in Geneious (version 2020.1.2) with the presence of both linker and LTR sequences in.sam files aligned to the domestic cat genome. Visual confirmation in Geneious revealed that between cats, the starting positions of integration sites were not always identical, and sites were considered the same a priori if they shared an integration site difference < 200 bp. Polarity of the LTR integration was determined based on the directionality of the Poisson distribution as the 5ʹ-LTR start site will appear as a blunt end given the presence of the LTR-primer site. The baited enrichment methods described above resulted in the capture of integration sites using the 5ʹ-LTR; however, it also captured enFeLV-env sequences as the 3ʹ LTR flanks the enFeLV-gene. Integration sites were removed if the adjacent genomic regions mapped to enFeLV-*env* genes as these regions are set in the reference genome and thus may not represent the true integration site in the specific cats sequenced. As a consequence, this effectively makes it impossible to discern solo-LTRs from full-length enFeLV LTRs, without further characterization of each integration site. Chromosomal integration sites were visually identified by scanning the annotated feline genome for genes 1 Mbp upstream and downstream of the integration sites in Geneious.

The difference between number of total and unique integration sites between the three cat cohorts was measured for statistical significance using a non-parametric Kruskall-Wallis test, following a Kolmogorov–Smirnov normality test in Prism v8. Analyses were performed examining statistical significance with and without one specific outlying datapoint (one SPF cat with uncharacteristically large number of integration sites).

### Bioinformatics analysis: gene expression analyses

Raw read sequencing quality was assessed using FastQC v. 0.11.9 and multiQC v. 1.12 [1], Ewels, Magnusson, Lundin and Kaller, 2016). Reads were trimmed to remove standard Illumina adapters using a stringency of 1 and were retained with a minimum length of 36, a minimum Phred score of 5, and an error rate of 0.1 using Trimgalore v. 0.6.5 [23]. Following trimming, we confirmed quality and adapter removal via FastQC and multiQC.

Prior to alignment and quantification, we concatenated domestic cat (FelCat9.0) and FeLV (ViralProj14686) genomes to capture exogenous and endogenous expression in tandem. We aligned trimmed reads to the concatenated FeLV-domestic cat genome using STAR v. 2.7.9a with the parameters “–outFilterMismatchNmax 3 –outFilterType BySJout –outFilterIntronMotifs RemoveNoncanonicalUnannotated” to remove reads with more than three mismatches, low confidence splice junctions, and unannotated noncanonical junctions [15]. We performed alignment using “–quantMode TranscriptomeSAM GeneCounts” to generate gene-level counts for all uniquely aligned reads.

To investigate gene expression among treatments, pairwise differential expression tests were performed using edgeR v. 3.23.3 [37]. We retained genes with expression of ≥ 1 counts per million (CPM) in at least 25% of all samples analyzed and normalized samples according to library size. Following filtering and confirmation of absence of outliers based on PCA, we estimated common and trended dispersion and fit generalized linear models. We performed likelihood ratio tests on pairwise comparisons of interest and considered genes with a false discovery rate (FDR) corrected *p*-value of 0.05 and log-fold change in expression of ≥ 1 and ≤ − 1 to be significant. We compared expression between uninfected fibroblasts and uninfected PBMCs, uninfected and infected fibroblasts, and among PBMCs with and without LTR integration sites. We further tested for significantly different expression among genes within promoter and enhancer distances from LTR integration sites, immediately flanking up to 1kb or up to 1Mb from the integration site, respectively. To clarify patterns of expression between uninfected fibroblasts and PBMCs, we performed overrepresentation analyses in Panther v. 17 with a Fisher’s t-test and Bonferroni correction [31].

### CRFK cell culture and fluorescent in situ hybridization (FISH)

To support that loci detected by NGS analysis represented a normal distribution of enFeLV integrations, we conducted FISH on the feline-derived cell line Crandall Reese Feline Kidney (CRFK), which were determined to have 7 copies of full-length enFeLV provirus and 63 enFeLV-LTRs [11]. CRFK and puma (*Puma* concolor) fibroblasts were cultured. Puma full skin biopsies were collected by Colorado Parks and Wildlife and primary fibroblasts were isolated using methods previously reported [11]. Forty nucleotide FeLV probes were designed against the entire 8.5 kb enFeLV proviral genome (Genbank Accession number: AY364318; KromaTid, Fort Collins CO). Actively dividing CRFK and puma cells were treated with 0.1 ug/mL colcemid and incubated for 4 h before metaphase spreads were fixed to slides. After a series of dehydration steps, 30 µL of enFeLV FISH probes were applied to the slide, followed by Dapi, and then visualized with a Coolsnap ES^2^ camera and running MetaVue Imaging System software (Molecular Devices, San Jose, CA). Telomere-FISH was used as a positive control for the FISH protocol following previously reported methods (telomere probes generously provided by Dr. Susan Bailey; [29]).

## Results

### Integration site identification

**Fig. 2:**
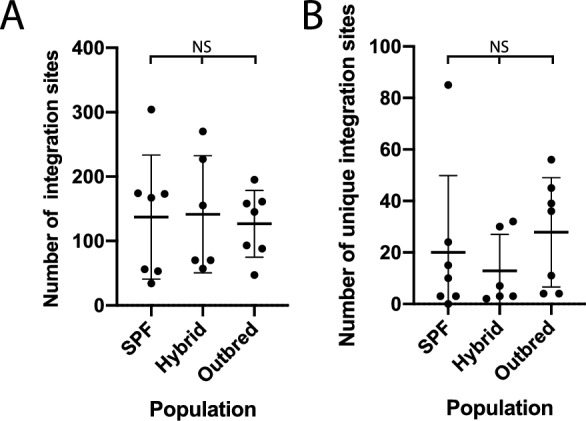
The source of tested cats had an impact on the location, but not the number of integration sites. **A** Cats, regardless of their source, had on average the same number of integration sites (Kruskall-Wallis; NS—p = 0.194) (**B**). Cats from an outbred group trended toward an average of greater number of unique integration sites compared to SPF cats (Kruskall-Wallis; NS—p = 0.081)

Among the 20 domestic cats deep-sequenced using a baited-PCR enrichment approach, we identified a total of 3,274 total integration sites that achieved at least 100 × read depth and were at least 100 nucleotides long (Fig. 1B). After removing enFeLV-*env-*associated integration sites. 2,704 integration sites remained among all 20 cats represented a total of 765 unique integration sites (Fig. 1B). Four hundred and twelve of the 765 unique sites (53.9%) were exclusive to one individual cat (Fig. 1B, Additional file 2: Figure S2, Table 1). The number of total integration sites among populations was not statistically different (Fig. 2A). The number of unique integration sites approached statistical significance when one outlier cat with an uncharacteristically large number (85) of unique integration sites was removed (included, Kruskall-Wallis adjusted p = 0.194; excluded, ANOVA adjusted p = 0.081; Fig. 2B).

**Table 1:** Summary of integrations and demographic data for individual cats of all three populations

Cat ID	Number of total integrations	Number of unique integrations	enFeLV-LTR copy number by qPCR	Sex
156	270	30	26	Male
178	277	32	47	Male
260	155	7	25	Female
282	57	3	60	Male
369	70	2	29	Female
377	70	3	42	Male
4438	174	24		Female
4460	173	15		Female
4474	167	10		Female
4501	304	85	36	Male
4504	53	3	121	Male
4510	34	3	89	Male
4520	56	0		Male
DC1	158	56	67	Male
DC2	161	45	57	Male
DC4	195	39	32	Female
DC6	88	4	57	Male
× 2654	93	11	74	Male
× 2656	145	37	62	Male
× 2657	47	4	49	Male

Integration sites were distributed across all chromosomes (Additional file 3: Figure S3). Three integrations sites were identified in all 20 cats and 87 integration sites were present in at least 10 individuals (Additional file 3: Figure S3; Additional file 7: Table S1).

Pairwise comparisons of individuals within each population showed that in populations with more closely related individuals, the number of shared integrations sites was greater, but not uniformly so (Additional file 1: Figure S1B-D). Population 1 had a range of 18 to 127 integrations. Population 2 had a range of 36 to 156. Population 3 had a range of 21 to 78.

Eighty-one integration sites were represented in 10 or more individuals in autosomal chromosomes and were within 1MB of 947 protein-coding genes (Additional file 8: Table S2). Five LTR integration sites (chrA2:7,199,456, chrA3:3,320,238, chrA3:77,888,994, chrD1:94,144,838, and chrF1:53,355,424) were not in close proximity to any identified coding regions. Six integrations were identified on the X chromosome; we were unable to identify any sites on the Y chromosome other than enFeLV-*env*. Only 24 genes were associated with the three consensus LTR integration sites found in all 20 cats (chrA3:3,320,238; chrB2:55,690,560; chrB2:146,684,232) (Additional file 7: Table S1). One LTR integration site in eight cats was found 599,886 bp from APOBEC1, a potent anti-retroviral protein (chrB4: 42584509) (Additional file 7: Table S1 and Additional file 7: Table S2).

enFeLV-LTR and full-length enFeLV integration distributions were validated by FISH. Telomere-FISH was used as a positive control (Additional file 4: Figure S4A). Hybridization for FeLV in CrFK cells is located across the entire genome (centromeres, telomeres, p-arms, and q-arms (Additional file 4: Figure S4B). FeLV did not hybridize in puma cells which lack enFeLV (Additional file 4: Figure S4C).

### RNA-sequencing and cell-specific gene expression

RNA from FeLV-uninfected and infected fibroblasts and PBMCs were quantified in order to detect alterations in host cell transcription post-infection. FeLV infection was confirmed as previously reported in [11]. RNA quality was poor in one uninfected and infected fibroblast pair leaving four sets for analysis. We retained 1.43 billion reads following trimming with a mean of 53.1 million reads per sample. Uninfected fibroblasts and PBMCs significantly differentially expressed 9,594 genes, equivalent to 60% of all genes tested. Functional enrichment analysis revealed enrichment for biological processes commonly associated with each cell class. We found 146 significantly enriched GO terms associated genes with increased expression in PBMCs, including antigen processing and presentation of peptide antigen, defense against bacterium, cell chemotaxis, and inflammatory response (Additional file 9: Table S3). Conversely, genes significantly upregulated by fibroblasts were enriched for 89 GO terms, including extracellular matrix organization, cell adhesion, and cell development (Additional file 10: Table S4).

**Fig. 3:**
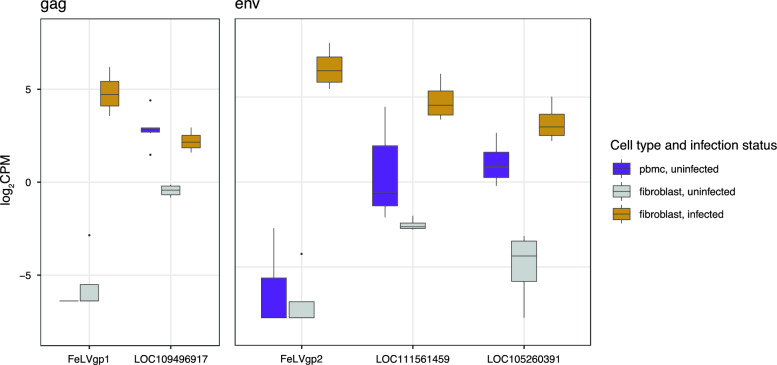
FeLV ortholog expression according to cell type and FeLV infection status. Evaluation of significantly differentially expressed genes in FeLV-negative PBMCs and fibroblasts, and FeLV-positive fibroblasts. Expression is quantified as the log_2_ value of counts per million mapped reads (CPM). Significance was determined via likelihood ratio tests with Benjamini–Hochberg to control for false discovery rate

Fibroblast gene expression was driven more by sample identity than by FeLV infection status (Additional file 5: Figure S5). Five genes were significantly differentially expressed when comparing uninfected and infected fibroblasts (Additional file 11: Table S5). Among these five genes, two were exogenous FeLV genes encoding the gag-pro-pol and env polyproteins (gene IDs: FeLVgp1 and FeLVgp2, respectively), and three were *Felis catus* genes encoding uncharacterized proteins (B4: LOC109496917, 85235918; B4: LOC111561459, 85238507; D3: LOC105260391, 73281). These three genes had high homology to exogenous *gag-pro-pol* and *env* genes based on blastn searches (Additional file 11: Table S5). Two of these genes were within promoter distance (up to 1kb downstream) of the nearest identified LTR integration site, while one was within enhancer distance (Additional file 11: Table S5). In all cases, gene transcription (*gag-pro-pol* and *env*) was upregulated during FeLV infection, and exogenous genes were more strongly upregulated than their endogenous orthologs (Fig. 3, Additional file 11: Table S5). Two of three endogenous genes were also significantly upregulated in uninfected PBMCs compared to uninfected fibroblasts, indicating cell-specific variation in baseline expression (LOC105260391, logFC 4.47, FDR-corrected *p*-value 1.11 × 10^–8^, and LOC109496917, logFC 3.53, FDR-corrected p-value 2.20 × 10^–10^) (Fig. 3).

### Gene expression responses to LTR integration presence/absence

Gene expression analyses were performed on non-FeLV-infected domestic cat fibroblasts and PBMCs while overlaying enFeLV-integrations sites to specifically evaluate baseline enFeLV-LTR-governed changes in gene transcription. We identified twelve genes within promoter distance (up to 1kb downstream) of LTR integration sites and 407 genes within enhancer distance of LTR integration sites that were shared by three or more of our samples. When we analyzed expression of genes within promoter or enhancer distance from LTR integration sites in a subset of at least three cats from the six PBMC samples evaluated, we found no significant differences in gene expression relative to presence or absence of LTR (Additional file 12: Table S6).

## Discussion

Endogenous retroviral elements influence host genome evolution and exogenous retroviral disease outcomes [10]. Here, we find that the FeLV-derived retrotransposome is highly variable among individual cats, consistent with findings in other systems [10, 30]. Crucially, our results demonstrate that the domestic cat transposome varies with respect to integration copy number and the majority of these sites are unique to an individual cat. This challenges the idea that while retrotransposition occurs leading to new integrations, the vast majority of these integration sites are inherited in a Mendelian fashion. While LTR elements may influence gene transcription [19], our findings provide additional evidence that enFeLV-LTRs do not function as promoter and enhancers [18], though a larger study with additional individuals may help identify individual LTR effects. More importantly, our investigation into the enFeLV-transposome reveals substantial variation, even among highly related individuals. We found that the number of enFeLV integrations identified by qPCR and NGS was incongruent. We believe this is related to the fact that qPCR involves two enFeLV-specific primers and the NGS enrichment step only requires one, it is possible that mutations in the 5ʹ primer used for the enFeLV-LTR may have underestimated the true number of integrations.

*The FeLV-transposome is individually unique.* More than half of the identified 765 LTR insertion sites were found in only one of 20 individuals (Additional file 7: Table S1); the vast majority of integration sites were found in four or fewer animals (Additional file 2: Figure S2). This suggests that LTR integration/retrotransposition is a dynamic process that results in a unique genetic map in each individual (Additional file 6: Figure S6). The presence of integration sites on all chromosomes was expected, as proviral integration is generally considered a random process during both retrotransposition and exogenous retroviral infection [25, 41]. Once established as an endogenous provirus, integration site fidelity is fairly well-conserved in retroviruses compared to other viral families [44], though the variation that exists in closely related populations may challenge this canon. This may suggest that the presence of individual integration sites may be governed by different processes.

Conserved proviral integrations may provide beneficial regulation of proximal host genes that result in selective advantage. For example, MuLV-LTR within the intronic sequence of mouse APOBEC3 promotes antiviral activity and is positively selected [38]. The presence of sequences derived from the xenotropic-MuLV is variable between laboratory strains as well as distinct house mouse (*Mus musculis)* subspecies and those that harbor the intronic MuLV-LTR sequence have increased transcription of mouse APOBEC3. The human retroviral literature has identified numerous human endogenous retroviral elements that have participated in critical immunomodulatory functions (summarized in [20], which may drive direct or indirect positive selection of these sites.

Three LTR integration sites were identified in all 20 cats. These loci may be the result of an ancient evolutionary history, the target of a selective advantage, or the beneficiary of linked regions under selection. These integrations could represent solo-LTRs or the 5ʹ-LTR of a full length enFeLV*.* One of the sites (chrA3:3,320,238) was not associated with any genes, but the other two sites (chrB2:55,690,560 and chrB2:146,684,232) were within promoter or enhancer distance of 24 genes. Notable associated genes include superoxide dismutase 2, which is important for hydrolyzation of reactive oxygen species, enzymes important in general cellular homeostasis (i.e., Map-3-kinase, Acetyl co-A acetyltransferase, Wild type 1 associate protein), and hypocretin receptor 2 involved in centrally regulating feeding behavior.

Solo LTR integration sites represent the culmination of integration and retrotransposition events, which are believed to be largely inherited [3]. Thus, we predicted the number of shared integration sites would be highest in cats within the inbred population (population 1), followed by the closed population (2), with the largest number of unique integration sites in the outbred population (3). While individuals within the outbred population had the smallest number of shared integration sites, we did not observe statistical difference among populations, and we observed substantial solo-LTR insertion site variation (Fig. 2, Additional file 1: Figure S1). As we do not have records of the full lineage even within the inbred population, we are unable to fully assess the patterns of inheritance from this twenty-cat study.

Male cats harbor more enFeLV-LTR copy numbers compared to females. In previous work, we identified that higher enFeLV-LTR copy number load is associated with resistance to exogenous FeLV progression [34]; however, identification of integration sites in the Y-chromosome was hindered by available annotated Y-chromosomes. The domestic cat Y-chromosome gene map was constructed independently from the rest of the feline whole genome-sequencing project [24] and consists of a large percentage of mobile elements that do not undergo recombination. Thus, the annotated Y-chromosome available for comparison (Accession number KP081775) is not representative of all Y-chromosomes. We mapped enFeLV-*env* in two to three loci on the Y-chromosome, indicating we had properly indexed the Y-chromosome for our bioinformatic approach, but were unable to map LTRs in this chromosome with the available reference data, and did not document higher integration site numbers in male cats because of these technical limitations (Table 1).

The conserved sites provide a tempting case example for possible positive evolutionary pressure; however, the vast majority of sites were shared by a small subset of individuals, which may represent alternative hypotheses of maintenance within the genome. If LTRs are no longer active and they exist in functionally inert regions of the genome, they may be under neutral evolutionary pressures where the integration does not affect the animal’s fitness. Whereas, integrations of LTRs that disrupt gene function may be under purifying selection. RNA sequencing data superimposed on integration site data discussed below helps to determine functionality.

To better evaluate functional roles of enFeLV-LTR integration sites, we evaluated their influence on transcription by identifying coding regions of the cat genome 1Mbp of each integration, as this distance is generally accepted as the maximal distance for LTR enhancer function [28, 33]. A small proportion of integration sites (10 of 81 integration sites present in ten or more cats) were not proximal to any annotated domestic cat genes. Among genes that were within enhancer distance, many gene classes were represented, including potent gene regulators (i.e., zinc finger genes) and anti-viral genes (i.e., APOBEC1). Our analysis to associate gene transcription with LTR insertion site did not detect significant changes in expression. This analysis was conducted from PBMCs from 6 cats in population 1 and fibroblast cultures from 4 cats in population 3. This low number of unreplicated samples likely limited statistical power of this preliminary analysis. It is possible that subtle shifts in gene expression among LTR-proximal genes does occur, which could be detected in studies specifically designed to assess this phenotype. Future studies should thus investigate gene expression variation among larger cohorts of cats harboring specific LTR integration sites of interest.

In vitro *FeLV infection increases expression of both exogenous and endogenous FeLV transcripts in fibroblasts.* Because the STAR aligner maps transcripts uniquely to one gene and we concatenated the FeLV and domestic cat genomes, we were able to quantify endogenous and exogenous FeLV transcription simultaneously. Five transcripts were significantly upregulated in FeLV-infected fibroblasts, which mapped to either enFeLV or exFeLV genes. As expected, exogenous *env* and *gag-pol-pro* polyprotein gene expression increased dramatically following exFeLV infection (879 and 1687-fold). More surprisingly, we documented relatively higher gene expression of enFeLV genes (7 to 85-fold). The baseline expression levels of two endogenous orthologs in uninfected PBMCs were relatively higher to uninfected fibroblasts in infected relative to uninfected fibroblasts., indicating that expression may vary according to cell type, similar to previous reports [9]. While other solo-LTRs were identified in close proximity to these sites, LTRs associated with enFeLV provirus would presumably be the effective promoters for these loci due to proximity. Importantly, we did not investigate the effects of infection on generation of miRNA, which we propose as the mechanism of viral restriction in previous reports [9]. We further acknowledge that limiting our gene expression quantification to only uniquely mapped reads may underestimate expression of gag-pol, which may be indistinguishable endogenously and exogenously. The upregulation we have identified warrants further analysis with larger sample sizes to determine mechanism of action and cell specificity, and this transcriptional response is a target for ongoing research.

## Conclusion

Our understanding of how endogenous retroviral elements influence host genomes has advanced considerably from the misguided notion of their existence as “junk DNA.” While the preponderance of ERVs and solo-LTRs are likely inherited, our work demonstrates that even in a closed population with a great degree of inbreeding, the individual FeLV-derived retrotransposome variation is substantial. While we provide little evidence that this variation alone may contribute to anti-exogenous FeLV infection, we provide a baseline for further inquiry into understanding the dynamic environment of endogenous retroviral retrotransposition. Furthermore, the lack of evidence of individual integration site effects on functional genomics may provide credence to our previous hypothesis that RNA interference may be a mechanism of endogenous FeLV-mediated restriction to exogenous FeLV infection.

### Supplementary Information

**Additional file 1: Figure S1.** A) Known matrilinear lineage of population 1 show relatedness of animals. Solid lines represent the individuals included in this study. Queens are denoted in squares and toms are denoted in circles. B) Pairwise comparisons between individuals in population 1 show the number of shared integration sites between two cats with a range of 18 to 127 integrations. C) Pairwise comparisons between individuals in population 2 show the number of shared integration sites between two cats with a range of 36 to 156. D) Pairwise comparisons between individuals in population 3 the number of shared integration sites between two cats with a range of 21 to 78.

**Additional file 2: Figure S2.** Number of enFeLV-LTR integration sites varies among cats with the majority of integration sites found in few individuals.

**Additional file 3: Figure S3.** Seven hundred sixty-five enFeLV-LTR integration sites are distributed across all chromosomes of the domestic cat genome. Only three sites found in chromosomes A3 and B2 are shared by all 20 cats. Very few non-unique integration sites are made up of cats solely from one cohort.

**Additional file 4: Figure S4.** Fluorescent *in situ *hybridization allows for the visualization of specific genomic elements in Crandall Reese Feline Kidney metaphase spreads. A) Probes specific for telomeres can be found at both ends of each chromosome. B) Probes specific for enFeLV appear to hybridize along the majority of chromosomes. The majority of signals are punctate, possibly indicating solo-LTRs, while some larger signals may indicate full-length enFeLV. Alternatively, greater signal may represent multiple integration sites in close proximity. C) Puma cells lack enFeLV and as such do not show signal to enFeLV probes.

**Additional file 5: Figure S5.** Principal components analysis of all samples included in gene expression analyses.

**Additional file 6: Figure S6.** Nineteen LTR integrations can be found on chromosome F2. Three integrations are found in at least 10 cats. Sixteen of the 19 integration sites are found in fewer than 10 cats. Representative cats from each population are provided (Cat 4460 – SPF; Cat 178 – hybrid; Cat DC1 – outbred) as examples of the diversity in individual cats.

**Additional file 7: Table S1.** Curated and compiled list of all integration sites separated by individual and population (1 = most inbred; 2 = less inbred; 3 = outbred).

**Additional file 8: Table S2.** 960 protein-coding genes are associated with all 80 integration sites found in at least ten individuals.

**Additional file 9: Table S3.** Overrepresented biological process GO terms in PBMCs relative fibroblasts. Analyses performed via Panther.

**Additional file 10: Table S4.** Overrepresented biological process GO terms in FeLV-positive relative to FeLV-negative fibroblasts. Analyses performed via Panther.

**Additional file 11: Table S5.** Significantly differentially expressed genes in FeLV-negative and FeLV-positive fibroblasts.

**Additional file 12: Table S6.** Genes within promoter and enhancer distance of LTR insertions shared by three or more PBMC samples.

## Data Availability

The datasets generated and analysed during the current study are available in the SRA, BioProject: PRJNA750528.
