# Supplementary figures and images for: Endogenous feline leukemia virus long terminal repeat integration site diversity is highly variable in related and unrelated domestic cats
Source: Retrovirology. 2024 Feb 12;21:3. doi: 10.1186/s12977-024-00635-0 (PMC10863107; doi:10.1186/s12977-024-00635-0)

A

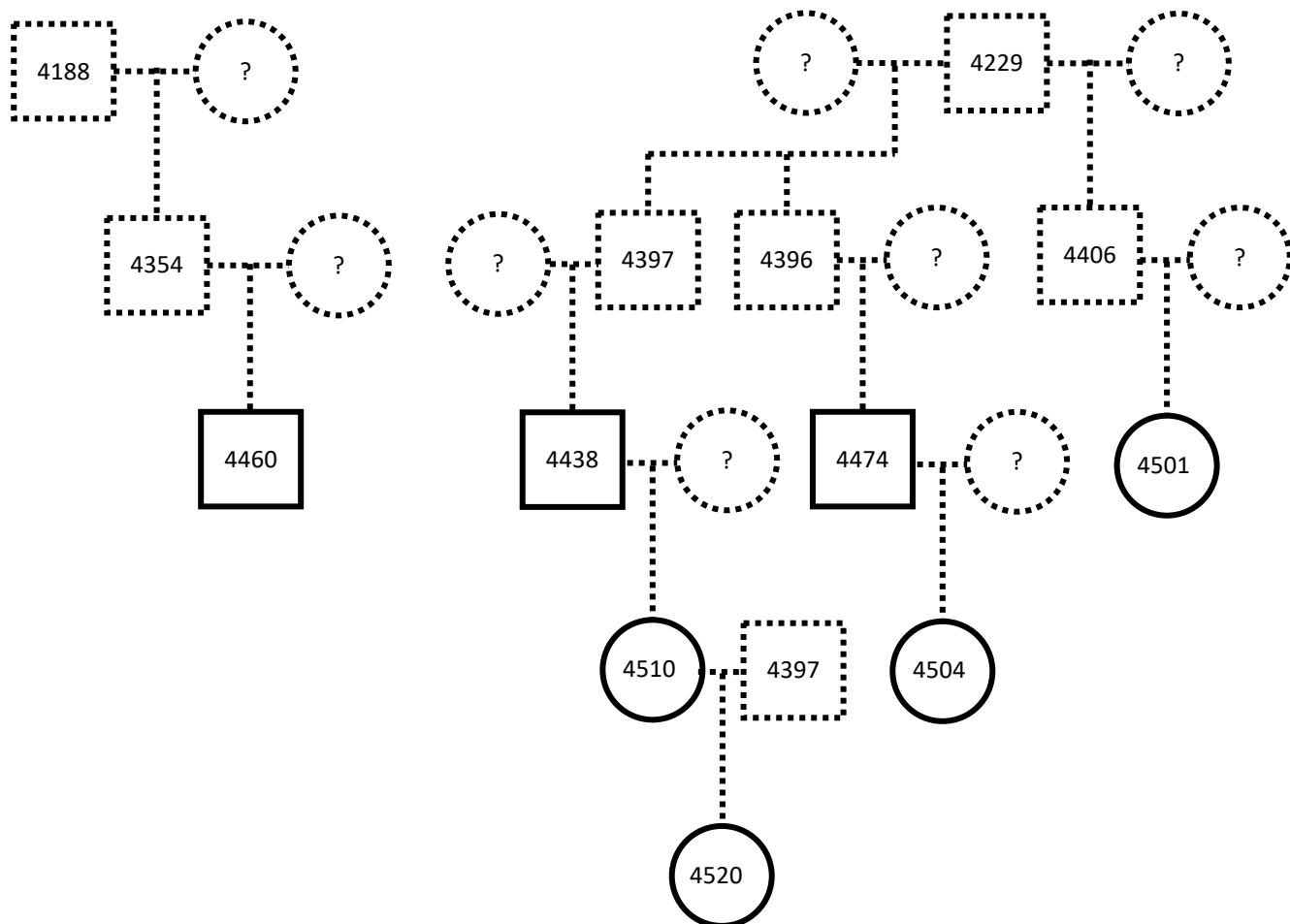

B

|      |      |      |      |      |      |      |
|------|------|------|------|------|------|------|
| 4460 | 108  |      |      |      |      |      |
| 4474 | 105  | 107  |      |      |      |      |
| 4510 | 122  | 127  | 124  |      |      |      |
| 4504 | 20   | 22   | 22   | 20   |      |      |
| 4501 | 36   | 37   | 37   | 35   | 22   |      |
| 4520 | 34   | 41   | 38   | 39   | 29   | 18   |
|      | 4438 | 4460 | 4474 | 4510 | 4504 | 4501 |

C

|     |     |     |     |     |     |  |
|-----|-----|-----|-----|-----|-----|--|
| 178 | 156 |     |     |     |     |  |
| 260 | 125 | 98  |     |     |     |  |
| 282 | 42  | 43  | 41  |     |     |  |
| 369 | 46  | 49  | 42  | 36  |     |  |
| 377 | 53  | 60  | 42  | 40  | 42  |  |
|     | 156 | 178 | 260 | 282 | 369 |  |

D

|       |     |     |     |     |       |       |
|-------|-----|-----|-----|-----|-------|-------|
| DC2   | 72  |     |     |     |       |       |
| DC4   | 72  | 84  |     |     |       |       |
| DC6   | 49  | 57  | 62  |     |       |       |
| x2654 | 21  | 27  | 29  | 28  |       |       |
| x2656 | 45  | 47  | 60  | 48  | 33    |       |
| x2657 | 66  | 78  | 74  | 53  | 27    | 46    |
|       | DC1 | DC2 | DC4 | DC6 | x2654 | x2656 |

Supplement: Supplementary file 1 — Additional file 1: Figure S1. A) Known matrilinear lineage of population 1 show relatedness of animals. Solid lines represent the individuals included in this study. Queens are denoted in squares and toms are denoted in circles. B) Pairwise comparisons between individuals in population 1 show the number of shared integration sites between two cats with a range of 18 to 127 integrations. C) Pairwise comparisons between individuals in population 2 show the number of shared integration sites between two cats with a range of 36 to 156. D) Pairwise comparisons between individuals in population 3 the number of shared integration sites between two cats with a range of 21 to 78. [file 12977_2024_635_MOESM1_ESM.pdf]

Number of Instances

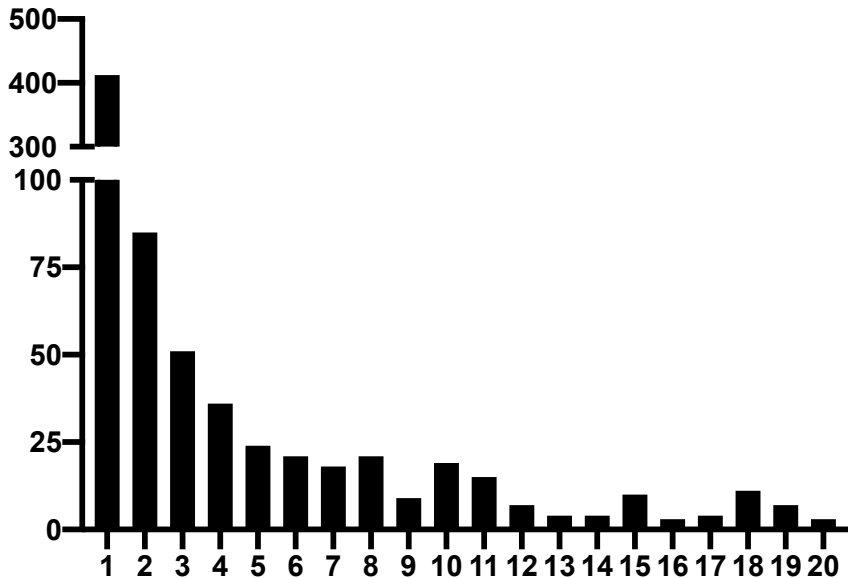

Number of cats harboring specific integration

Supplement: Supplementary file 2 — Additional file 2: Figure S2. Number of enFeLV-LTR integration sites varies among cats with the majority of integration sites found in few individuals. [file 12977_2024_635_MOESM2_ESM.pdf]

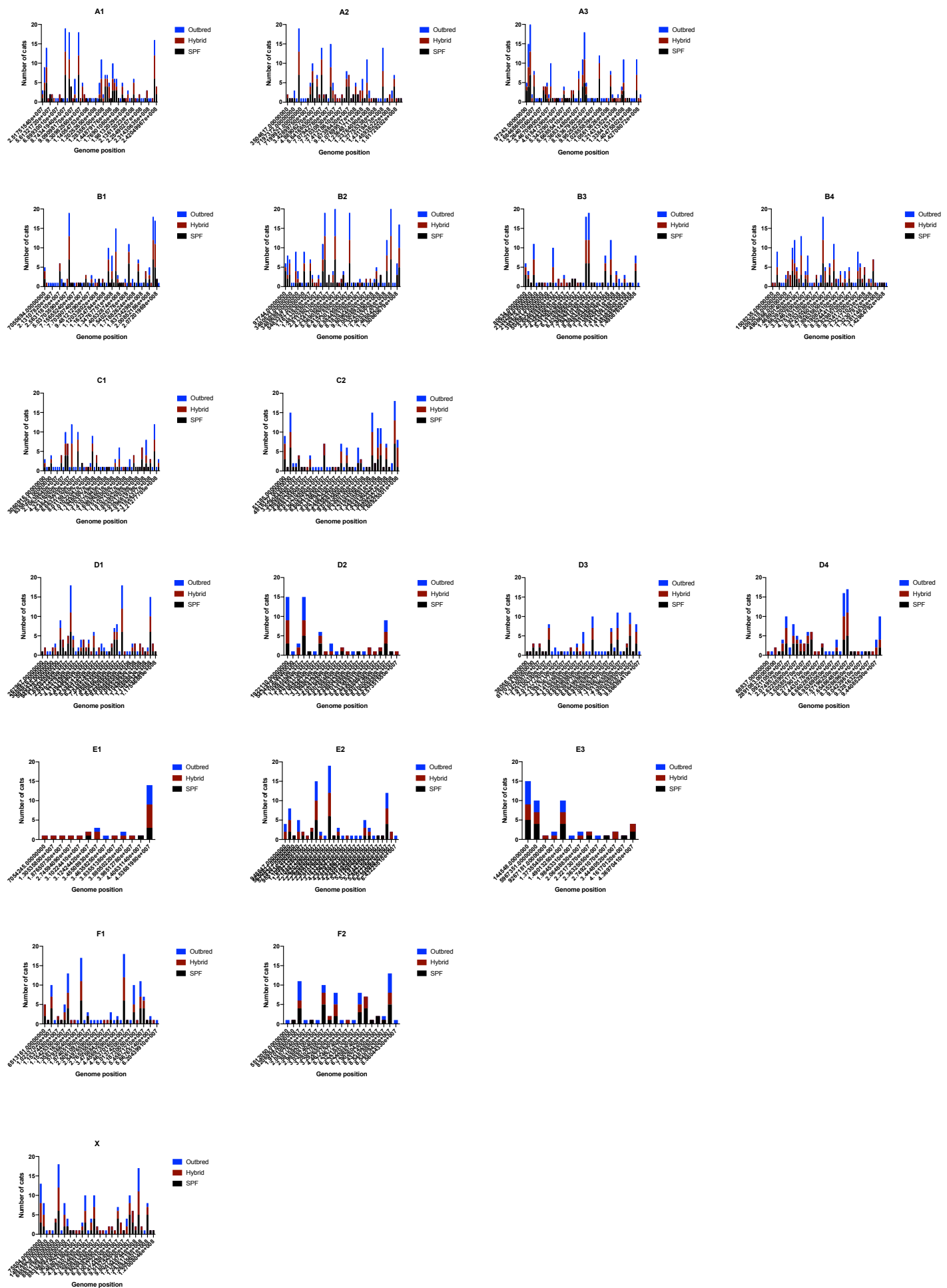

Supplement: Supplementary file 3 — Additional file 3: Figure S3. Seven hundred sixty-five enFeLV-LTR integration sites are distributed across all chromosomes of the domestic cat genome. Only three sites found in chromosomes A3 and B2 are shared by all 20 cats. Very few non-unique integration sites are made up of cats solely from one cohort. [file 12977_2024_635_MOESM3_ESM.pdf]

Dapi  
Telomere

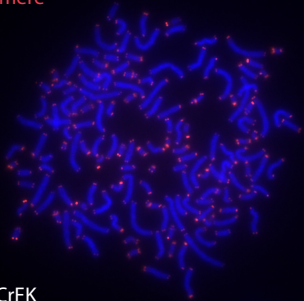

A: CrFK

Dapi  
enFeLV

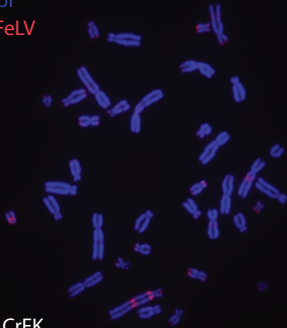

B: CrFK

Dapi  
enFeLV

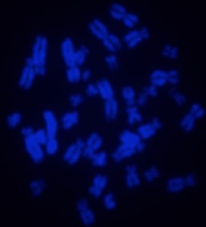

C: Puma

Supplement: Supplementary file 4 — Additional file 4: Figure S4. Fluorescent in situ hybridization allows for the visualization of specific genomic elements in Crandall Reese Feline Kidney metaphase spreads. A) Probes specific for telomeres can be found at both ends of each chromosome. B) Probes specific for enFeLV appear to hybridize along the majority of chromosomes. The majority of signals are punctate, possibly indicating solo-LTRs, while some larger signals may indicate full-length enFeLV. Alternatively, greater signal may represent multiple integration sites in close proximity. C) Puma cells lack enFeLV and as such do not show signal to enFeLV probes. [file 12977_2024_635_MOESM4_ESM.pdf]

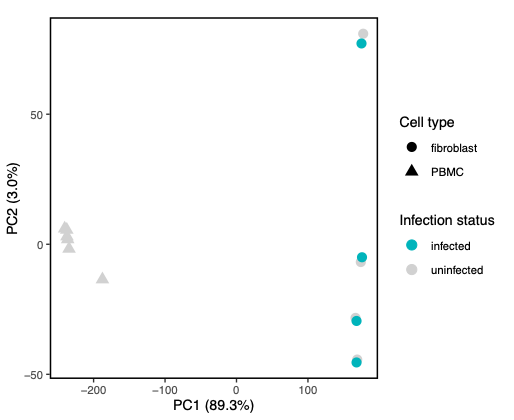

Supplement: Supplementary file 5 — Additional file 5: Figure S5. Principal components analysis of all samples included in gene expression analyses. [file 12977_2024_635_MOESM5_ESM.png]

# Chromosome

## Integration sites in < 10 cats

**F2**

## Integration sites in 10+ cats

**Cat 4460**

**Cat 178**

**Cat DC1**

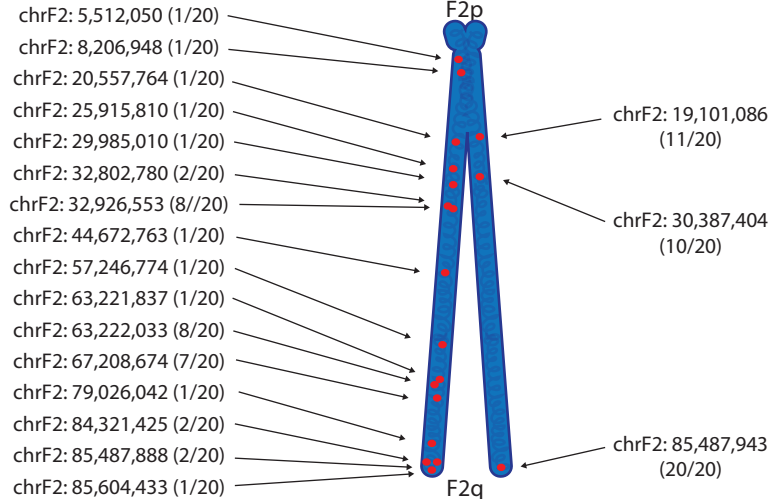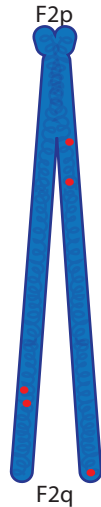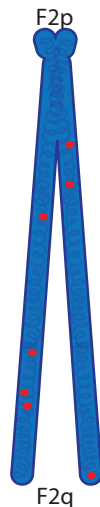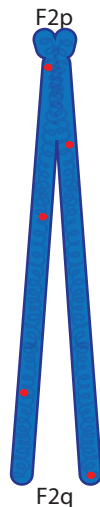

Supplement: Supplementary file 6 — Additional file 6: Figure S6. Nineteen LTR integrations can be found on chromosome F2. Three integrations are found in at least 10 cats. Sixteen of the 19 integration sites are found in fewer than 10 cats. Representative cats from each population are provided (Cat 4460 – SPF; Cat 178 – hybrid; Cat DC1 – outbred) as examples of the diversity in individual cats. [file 12977_2024_635_MOESM6_ESM.pdf]
