# Supplementary material for: Endogenous feline leukemia virus long terminal repeat integration site diversity is highly variable in related and unrelated domestic cats
Source: Retrovirology. 2024 Feb 12;21:3. doi: 10.1186/s12977-024-00635-0 (PMC10863107; doi:10.1186/s12977-024-00635-0)
Supplement: Supplementary file 7 — Additional file 7: Table S1. Curated and compiled list of all integration sites separated by individual and population (1 = most inbred; 2 = less inbred; 3 = outbred). [file 12977_2024_635_MOESM7_ESM.pdf]

Chromosome		start site	Population 1	Population 2	Population 3	Total Integration site
NC_018723	A1	25175154	1	1	1	3
NC_018723	A1	35302671	3	2	4	9
NC_018723	A1	37559850	5	4	5	14
NC_018723	A1	56132140	0	1	0	1
NC_018723	A1	56132172	2	0	0	2
NC_018723	A1	58001328	1	1	0	2
NC_018723	A1	60384921	0	1	0	1
NC_018723	A1	65829429	0	0	1	1
NC_018723	A1	68993001	0	0	1	1
NC_018723	A1	75673842	0	2	0	2
NC_018723	A1	84436185	1	0	0	1
NC_018723	A1	85439315	0	0	1	1
NC_018723	A1	87434194	7	6	6	19
NC_018723	A1	87434665	0	0	1	1
NC_018723	A1	87661908	6	5	7	18
NC_018723	A1	87734124	4	0	0	4
NC_018723	A1	90909817	0	1	0	1
NC_018723	A1	91818785	1	1	4	6
NC_018723	A1	91985765	0	0	1	1
NC_018723	A1	91986082	7	5	6	18
NC_018723	A1	93098256	0	1	0	1
NC_018723	A1	95328510	1	3	1	5
NC_018723	A1	101976153	0	2	0	2
NC_018723	A1	107606821	1	0	0	1
NC_018723	A1	114054753	0	0	1	1
NC_018723	A1	115145561	1	0	0	1
NC_018723	A1	115750876	0	0	1	1
NC_018723	A1	118300344	0	0	1	1
NC_018723	A1	120252650	1	0	0	1
NC_018723	A1	120783226	0	0	1	1
NC_018723	A1	125566171	1	1	3	5
NC_018723	A1	125666538	1	5	5	11
NC_018723	A1	128356700	0	1	1	2
NC_018723	A1	140939893	4	2	1	7
NC_018723	A1	142477577	4	2	1	7
NC_018723	A1	143377630	1	3	1	5
NC_018723	A1	144879176	1	0	0	1
NC_018723	A1	154602427	3	3	4	10
NC_018723	A1	169659171	3	2	1	6
NC_018723	A1	171549806	3	1	2	6
NC_018723	A1	176901080	1	0	0	1
NC_018723	A1	178813499	0	0	1	1
NC_018723	A1	190088341	2	2	1	5
NC_018723	A1	194943338	1	0	0	1

NC_018723	A1	198132029	0	1	0	1
NC_018723	A1	208330102	0	2	1	3
NC_018723	A1	208330533	0	0	1	1
NC_018723	A1	211977644	1	0	0	1
NC_018723	A1	213612023	2	3	1	6
NC_018723	A1	218113147	0	1	0	1
NC_018723	A1	219816579	0	0	1	1
NC_018723	A1	222933422	1	1	0	2
NC_018723	A1	225490257	0	0	1	1
NC_018723	A1	226705592	0	0	1	1
NC_018723	A1	229002105	1	0	0	1
NC_018723	A1	231430603	1	2	0	3
NC_018723	A1	231430635	0	0	1	1
NC_018723	A1	232216649	0	0	1	1
NC_018723	A1	233479948	0	1	0	1
NC_018723	A1	241024241	6	6	4	16
NC_018723	A1	242049967	2	2	0	4
NC_018724	A2	3504617	1	1	0	2
NC_018724	A2	5364618	1	0	0	1
NC_018724	A2	5606481	1	0	0	1
NC_018724	A2	7197372	1	0	2	3
NC_018724	A2	7197775	0	0	1	1
NC_018724	A2	7199456	7	6	6	19
NC_018724	A2	7199921	0	0	1	1
NC_018724	A2	8268269	0	0	1	1
NC_018724	A2	21616066	0	0	1	1
NC_018724	A2	34289975	1	0	0	1
NC_018724	A2	45360947	2	2	1	5
NC_018724	A2	48890477	5	3	2	10
NC_018724	A2	48890627	1	0	0	1
NC_018724	A2	48895504	4	2	1	7
NC_018724	A2	56066368	1	1	0	2
NC_018724	A2	56416655	7	4	3	14
NC_018724	A2	62202135	1	1	0	2
NC_018724	A2	70794116	2	0	0	2
NC_018724	A2	70794278	0	0	1	1
NC_018724	A2	70794560	4	5	6	15
NC_018724	A2	72079985	1	2	2	5
NC_018724	A2	72103083	0	2	0	2
NC_018724	A2	72124204	0	1	0	1
NC_018724	A2	72785471	0	1	0	1
NC_018724	A2	74121861	1	1	0	2
NC_018724	A2	79638668	1	0	0	1
NC_018724	A2	84283614	4	2	2	8
NC_018724	A2	96738643	4	3	0	7

NC_018724	A2	102004343	1	0	0	1
NC_018724	A2	107699365	2	0	0	2
NC_018724	A2	107699517	2	2	1	5
NC_018724	A2	117826112	2	0	0	2
NC_018724	A2	119963132	0	3	0	3
NC_018724	A2	123648711	1	2	3	6
NC_018724	A2	123656642	1	0	0	1
NC_018724	A2	123657107	2	3	6	11
NC_018724	A2	123764090	0	1	2	3
NC_018724	A2	123932540	0	1	0	1
NC_018724	A2	124642691	1	0	0	1
NC_018724	A2	127755338	1	0	0	1
NC_018724	A2	132176754	0	0	1	1
NC_018724	A2	132490472	0	0	1	1
NC_018724	A2	143119473	4	4	6	14
NC_018724	A2	143119921	0	0	1	1
NC_018724	A2	144612530	1	0	0	1
NC_018724	A2	145936222	0	2	0	2
NC_018724	A2	158856465	0	1	2	3
NC_018724	A2	158856805	4	2	1	7
NC_018724	A2	161729202	0	1	0	1
NC_018724	A2	168115819	1	0	0	1
NC_018724	A2	168611521	1	0	0	1
NC_018725	A3	97243	3	1	1	5
NC_018725	A3	97444	4	5	6	15
NC_018725	A3	3320238	7	6	7	20
NC_018725	A3	9514111	0	1	1	2
NC_018725	A3	16846868	4	3	1	8
NC_018725	A3	17424743	0	0	1	1
NC_018725	A3	20678789	0	0	1	1
NC_018725	A3	21179848	1	0	0	1
NC_018725	A3	22640106	0	0	1	1
NC_018725	A3	25046463	3	1	0	4
NC_018725	A3	27718496	2	2	0	4
NC_018725	A3	30028779	1	3	1	5
NC_018725	A3	34670990	0	1	1	2
NC_018725	A3	38977157	1	3	6	10
NC_018725	A3	38977234	1	0	0	1
NC_018725	A3	39465000	0	1	0	1
NC_018725	A3	46184739	1	0	0	1
NC_018725	A3	47886252	1	0	0	1
NC_018725	A3	49092724	0	0	1	1
NC_018725	A3	53432450	0	1	0	1
NC_018725	A3	53432907	2	1	1	4
NC_018725	A3	54762771	1	0	0	1

NC_018725	A3	54762795	1	0	0	1
NC_018725	A3	54762797	1	0	0	1
NC_018725	A3	56685541	1	2	0	3
NC_018725	A3	66085002	2	1	0	3
NC_018725	A3	69560449	0	0	1	1
NC_018725	A3	72659605	0	0	1	1
NC_018725	A3	73683148	3	3	2	8
NC_018725	A3	75362750	0	0	1	1
NC_018725	A3	77888994	4	3	4	11
NC_018725	A3	77917349	5	6	7	18
NC_018725	A3	81636795	2	2	1	5
NC_018725	A3	88085665	0	0	1	1
NC_018725	A3	96497853	0	0	1	1
NC_018725	A3	97466570	1	0	0	1
NC_018725	A3	99525822	0	0	1	1
NC_018725	A3	99646336	0	0	1	1
NC_018725	A3	100633299	1	0	0	1
NC_018725	A3	102855515	6	4	2	12
NC_018725	A3	102855839	0	0	1	1
NC_018725	A3	102856017	0	0	1	1
NC_018725	A3	103136324	0	0	1	1
NC_018725	A3	105302481	0	1	0	1
NC_018725	A3	105613626	0	0	1	1
NC_018725	A3	107101889	4	3	1	8
NC_018725	A3	108613548	0	1	1	2
NC_018725	A3	121172692	1	0	0	1
NC_018725	A3	121431352	0	1	0	1
NC_018725	A3	122582842	0	1	1	2
NC_018725	A3	124634159	0	1	0	1
NC_018725	A3	133841542	2	1	1	4
NC_018725	A3	133841631	3	2	6	11
NC_018725	A3	135141495	0	1	0	1
NC_018725	A3	137732538	1	0	0	1
NC_018725	A3	139980037	1	0	0	1
NC_018725	A3	140475802	0	0	1	1
NC_018725	A3	141439190	0	0	1	1
NC_018725	A3	141732586	0	0	1	1
NC_018725	A3	142200005	3	4	4	11
NC_018725	A3	142708072	0	1	0	1
NC_018725	A3	142708171	0	1	1	2
NC_018726	B1	7050694	2	2	1	5
NC_018726	B1	10724663	0	1	0	1
NC_018726	B1	16952534	0	0	1	1
NC_018726	B1	17135834	0	0	1	1
NC_018726	B1	21310782	0	0	1	1

NC_018726	B1	22187112	0	0	1	1
NC_018726	B1	22594540	0	0	1	1
NC_018726	B1	23320252	0	0	1	1
NC_018726	B1	25631181	4	2	0	6
NC_018726	B1	38181490	0	0	2	2
NC_018726	B1	49423830	1	0	0	1
NC_018726	B1	49826510	0	0	1	1
NC_018726	B1	50370359	0	2	0	2
NC_018726	B1	67270589	7	6	6	19
NC_018726	B1	69028628	1	0	0	1
NC_018726	B1	69028673	1	0	0	1
NC_018726	B1	71565524	0	0	1	1
NC_018726	B1	74938306	0	1	0	1
NC_018726	B1	76741407	1	0	0	1
NC_018726	B1	76775981	1	0	0	1
NC_018726	B1	77039074	0	0	1	1
NC_018726	B1	77322679	1	0	0	1
NC_018726	B1	77767892	2	1	0	3
NC_018726	B1	91647664	1	0	0	1
NC_018726	B1	94812129	0	0	1	1
NC_018726	B1	95538792	1	1	1	3
NC_018726	B1	96127544	0	0	1	1
NC_018726	B1	107463004	0	1	1	2
NC_018726	B1	112033802	0	1	0	1
NC_018726	B1	115378502	2	0	0	2
NC_018726	B1	115378528	0	0	1	1
NC_018726	B1	115378531	1	1	0	2
NC_018726	B1	116234784	1	0	0	1
NC_018726	B1	121267358	0	0	1	1
NC_018726	B1	124950589	4	3	3	10
NC_018726	B1	130236584	1	0	0	1
NC_018726	B1	134767751	2	2	4	8
NC_018726	B1	136552127	1	0	0	1
NC_018726	B1	136552325	4	5	6	15
NC_018726	B1	137848886	1	1	1	3
NC_018726	B1	140532874	1	0	0	1
NC_018726	B1	142349651	1	0	0	1
NC_018726	B1	145769867	1	0	0	1
NC_018726	B1	152810687	0	1	1	2
NC_018726	B1	154025776	0	0	1	1
NC_018726	B1	156352163	6	3	2	11
NC_018726	B1	165135830	0	1	0	1
NC_018726	B1	169944830	0	0	2	2
NC_018726	B1	171882624	1	0	0	1
NC_018726	B1	178910305	0	0	1	1

NC_018726	B1	180649645	4	2	1	7
NC_018726	B1	180649753	0	0	1	1
NC_018726	B1	183734298	0	0	1	1
NC_018726	B1	187376932	1	0	0	1
NC_018726	B1	194819674	2	2	0	4
NC_018726	B1	195089406	0	0	1	1
NC_018726	B1	200852186	2	1	2	5
NC_018726	B1	201480233	0	1	0	1
NC_018726	B1	201827018	7	5	6	18
NC_018726	B1	207201855	5	6	6	17
NC_018726	B1	207201969	2	0	0	2
NC_018726	B1	207202022	0	0	1	1
NC_018727	B2	97744	4	1	1	6
NC_018727	B2	2325282	2	1	5	8
NC_018727	B2	2942171	3	3	1	7
NC_018727	B2	3480523	0	0	1	1
NC_018727	B2	3480687	0	0	1	1
NC_018727	B2	3487959	1	3	5	9
NC_018727	B2	3488689	1	1	2	4
NC_018727	B2	3488752	1	0	0	1
NC_018727	B2	3488833	0	0	1	1
NC_018727	B2	5483397	4	2	3	9
NC_018727	B2	9713529	0	0	1	1
NC_018727	B2	11412518	1	0	0	1
NC_018727	B2	11671183	4	2	1	7
NC_018727	B2	14821672	1	1	1	3
NC_018727	B2	14821811	0	1	0	1
NC_018727	B2	14822038	0	1	0	1
NC_018727	B2	15402052	0	2	1	3
NC_018727	B2	24664832	0	0	1	1
NC_018727	B2	28112801	4	3	4	11
NC_018727	B2	32150838	7	6	6	19
NC_018727	B2	42341667	1	0	0	1
NC_018727	B2	42341702	3	0	0	3
NC_018727	B2	43863962	1	0	0	1
NC_018727	B2	51248392	1	2	1	4
NC_018727	B2	55690560	7	6	7	20
NC_018727	B2	60828894	1	0	0	1
NC_018727	B2	64142644	0	1	0	1
NC_018727	B2	64142785	1	1	0	2
NC_018727	B2	64142810	3	0	1	4
NC_018727	B2	65092104	0	0	1	1
NC_018727	B2	65730122	1	0	0	1
NC_018727	B2	65730248	6	6	7	19
NC_018727	B2	68257172	0	0	1	1

NC_018727	B2	68844590	1	0	0	1
NC_018727	B2	71865692	0	0	1	1
NC_018727	B2	72357879	1	0	0	1
NC_018727	B2	81835128	0	1	1	2
NC_018727	B2	83060240	1	0	0	1
NC_018727	B2	93365180	0	0	1	1
NC_018727	B2	96096452	1	1	0	2
NC_018727	B2	99782901	0	0	1	1
NC_018727	B2	103833188	1	1	0	2
NC_018727	B2	105506933	0	0	1	1
NC_018727	B2	130597895	1	0	1	2
NC_018727	B2	131628700	2	2	1	5
NC_018727	B2	132431690	0	0	1	1
NC_018727	B2	132541169	3	0	0	3
NC_018727	B2	135453454	0	0	1	1
NC_018727	B2	140537726	0	0	1	1
NC_018727	B2	140538176	4	4	4	12
NC_018727	B2	146279928	1	0	0	1
NC_018727	B2	146684232	7	6	7	20
NC_018727	B2	148990418	0	0	1	1
NC_018727	B2	149248644	0	0	1	1
NC_018727	B2	150860679	3	0	3	6
NC_018727	B2	150861037	5	5	6	16
NC_018728	B3	80634	3	2	1	6
NC_018728	B3	80786	2	1	1	4
NC_018728	B3	2130299	1	0	0	1
NC_018728	B3	2130434	3	4	4	11
NC_018728	B3	2132975	0	0	1	1
NC_018728	B3	3045169	1	0	0	1
NC_018728	B3	3935621	1	0	0	1
NC_018728	B3	4116627	0	1	0	1
NC_018728	B3	5040633	0	0	1	1
NC_018728	B3	23545229	1	0	0	1
NC_018728	B3	25546595	1	4	5	10
NC_018728	B3	25547041	0	0	1	1
NC_018728	B3	28344450	0	2	0	2
NC_018728	B3	41701100	0	1	0	1
NC_018728	B3	42564061	0	2	1	3
NC_018728	B3	52016983	0	1	0	1
NC_018728	B3	53129024	1	0	0	1
NC_018728	B3	61170640	2	0	0	2
NC_018728	B3	61170886	2	0	0	2
NC_018728	B3	65607789	0	1	0	1
NC_018728	B3	66284566	1	0	0	1
NC_018728	B3	69459534	0	0	1	1

NC_018728	B3	74694912	6	6	6	18
NC_018728	B3	79470596	6	6	7	19
NC_018728	B3	79471015	1	0	0	1
NC_018728	B3	85418172	1	0	0	1
NC_018728	B3	85983591	0	0	1	1
NC_018728	B3	87333964	0	0	1	1
NC_018728	B3	93925448	1	0	0	1
NC_018728	B3	129403506	4	2	2	8
NC_018728	B3	131089636	0	0	1	1
NC_018728	B3	136800478	3	4	5	12
NC_018728	B3	139506471	0	0	1	1
NC_018728	B3	140527232	1	2	1	4
NC_018728	B3	141210919	0	0	2	2
NC_018728	B3	141211908	0	0	1	1
NC_018728	B3	141212380	1	1	1	3
NC_018728	B3	143163800	0	0	1	1
NC_018728	B3	145984520	1	0	0	1
NC_018728	B3	146896411	0	0	1	1
NC_018728	B3	149087602	4	2	2	8
NC_018728	B3	149569990	0	0	1	1
NC_018729	B4	1508235	0	1	0	1
NC_018729	B4	1508430	0	1	0	1
NC_018729	B4	1508834	3	2	4	9
NC_018729	B4	4093018	1	0	0	1
NC_018729	B4	4748814	0	0	1	1
NC_018729	B4	4908438	0	0	1	1
NC_018729	B4	4908666	1	1	1	3
NC_018729	B4	5654454	2	2	0	4
NC_018729	B4	12275158	0	3	0	3
NC_018729	B4	14689014	4	3	3	10
NC_018729	B4	24617796	3	3	6	12
NC_018729	B4	25275803	2	2	1	5
NC_018729	B4	26639208	1	1	0	2
NC_018729	B4	29973213	3	5	5	13
NC_018729	B4	31381515	0	0	1	1
NC_018729	B4	39346780	2	1	1	4
NC_018729	B4	42584509	1	2	5	8
NC_018729	B4	47519301	0	0	1	1
NC_018729	B4	47519318	0	0	1	1
NC_018729	B4	47519403	0	1	0	1
NC_018729	B4	53646388	2	1	0	3
NC_018729	B4	59253228	0	0	1	1
NC_018729	B4	59253464	1	0	2	3
NC_018729	B4	64351845	6	6	6	18
NC_018729	B4	64792601	1	3	1	5

NC_018729	B4	76428681	1	0	0	1
NC_018729	B4	77544578	3	2	1	6
NC_018729	B4	78963585	0	1	0	1
NC_018729	B4	79102334	4	3	4	11
NC_018729	B4	80282240	1	1	0	2
NC_018729	B4	81908241	0	0	2	2
NC_018729	B4	82998468	1	2	1	4
NC_018729	B4	84135225	0	1	0	1
NC_018729	B4	85244412	1	1	2	4
NC_018729	B4	85244974	0	0	1	1
NC_018729	B4	88298434	2	2	1	5
NC_018729	B4	88298570	1	0	0	1
NC_018729	B4	90710486	0	0	1	1
NC_018729	B4	93341224	0	0	1	1
NC_018729	B4	93341700	2	2	5	9
NC_018729	B4	95302586	2	3	1	6
NC_018729	B4	96989588	1	1	0	2
NC_018729	B4	122171023	3	2	0	5
NC_018729	B4	124657729	0	0	1	1
NC_018729	B4	131214486	1	1	0	2
NC_018729	B4	132307812	0	1	1	2
NC_018729	B4	132307818	4	3	0	7
NC_018729	B4	134532773	0	1	0	1
NC_018729	B4	138114838	0	0	1	1
NC_018729	B4	140212098	1	0	0	1
NC_018729	B4	142740711	1	0	0	1
NC_018729	B4	142984792	1	0	0	1
NC_018729	B4	142985026	0	0	1	1
NC_018730	C1	3060916	1	1	1	3
NC_018730	C1	3061385	0	0	1	1
NC_018730	C1	5892508	1	0	0	1
NC_018730	C1	6358251	2	1	1	4
NC_018730	C1	9373628	0	0	1	1
NC_018730	C1	18421936	0	0	1	1
NC_018730	C1	27063964	0	0	1	1
NC_018730	C1	31869360	0	0	1	1
NC_018730	C1	38114892	2	2	0	4
NC_018730	C1	43211608	0	0	1	1
NC_018730	C1	46392926	4	3	3	10
NC_018730	C1	53329819	4	3	0	7
NC_018730	C1	54647925	0	0	1	1
NC_018730	C1	58764761	1	6	5	12
NC_018730	C1	60037708	0	0	1	1
NC_018730	C1	66632715	0	0	1	1
NC_018730	C1	68452850	5	3	2	10

NC_018730	C1	76200154	0	0	1	1
NC_018730	C1	80115976	2	0	0	2
NC_018730	C1	90924920	0	0	1	1
NC_018730	C1	101020674	0	1	0	1
NC_018730	C1	101022047	1	0	0	1
NC_018730	C1	115436700	0	1	1	2
NC_018730	C1	115437078	5	2	2	9
NC_018730	C1	119465887	0	0	1	1
NC_018730	C1	127468099	2	2	0	4
NC_018730	C1	127766234	1	0	0	1
NC_018730	C1	137037749	0	0	1	1
NC_018730	C1	140834789	1	0	0	1
NC_018730	C1	140920852	0	0	1	1
NC_018730	C1	141075945	1	0	0	1
NC_018730	C1	150357324	1	0	0	1
NC_018730	C1	152589437	0	0	1	1
NC_018730	C1	169856601	0	0	1	1
NC_018730	C1	172607490	1	1	1	3
NC_018730	C1	172850890	1	0	0	1
NC_018730	C1	175128008	1	2	3	6
NC_018730	C1	176453213	0	0	1	1
NC_018730	C1	177745731	0	1	0	1
NC_018730	C1	181071037	1	0	0	1
NC_018730	C1	186626194	0	0	1	1
NC_018730	C1	188394950	1	0	2	3
NC_018730	C1	188395289	0	0	1	1
NC_018730	C1	198975553	2	2	0	4
NC_018730	C1	199546309	1	0	0	1
NC_018730	C1	203217039	1	0	0	1
NC_018730	C1	204366634	1	0	0	1
NC_018730	C1	207876207	3	3	0	6
NC_018730	C1	209661979	1	0	0	1
NC_018730	C1	209662084	2	2	4	8
NC_018730	C1	210578563	1	0	0	1
NC_018730	C1	211824377	2	1	0	3
NC_018730	C1	215632776	0	0	1	1
NC_018730	C1	219236324	5	3	4	12
NC_018730	C1	221277705	0	0	1	1
NC_018730	C1	221278199	1	1	1	3
NC_018731	C2	51385	3	4	2	9
NC_018731	C2	4815776	1	0	0	1
NC_018731	C2	4816127	6	4	5	15
NC_018731	C2	9436703	1	0	1	2
NC_018731	C2	35290644	1	0	1	2
NC_018731	C2	38330139	3	1	0	4

NC_018731	C2	38959025	1	0	0	1
NC_018731	C2	42606124	1	0	0	1
NC_018731	C2	44861356	0	1	0	1
NC_018731	C2	45047380	1	2	1	4
NC_018731	C2	50712102	0	0	1	1
NC_018731	C2	52908551	0	0	1	1
NC_018731	C2	55670506	0	0	1	1
NC_018731	C2	58656428	0	0	1	1
NC_018731	C2	66864743	4	3	0	7
NC_018731	C2	70625537	0	0	1	1
NC_018731	C2	73713665	0	0	1	1
NC_018731	C2	77372453	1	0	0	1
NC_018731	C2	80517941	0	1	0	1
NC_018731	C2	82465700	1	0	0	1
NC_018731	C2	82466254	2	3	2	7
NC_018731	C2	83355307	0	0	1	1
NC_018731	C2	83355810	2	2	2	6
NC_018731	C2	86890353	1	0	0	1
NC_018731	C2	90787560	1	0	0	1
NC_018731	C2	96529592	0	0	1	1
NC_018731	C2	96530075	2	0	4	6
NC_018731	C2	110715554	2	1	0	3
NC_018731	C2	112510300	0	0	1	1
NC_018731	C2	117648059	1	0	0	1
NC_018731	C2	131951056	1	0	0	1
NC_018731	C2	131951595	4	6	5	15
NC_018731	C2	134930581	2	0	0	2
NC_018731	C2	135744902	3	3	5	11
NC_018731	C2	143414052	4	3	4	11
NC_018731	C2	156830221	1	0	0	1
NC_018731	C2	158202327	3	3	1	7
NC_018731	C2	159342010	1	0	0	1
NC_018731	C2	160924278	0	0	2	2
NC_018731	C2	160924755	7	6	5	18
NC_018731	C2	160925501	1	5	2	8
NC_018732	D1	267967	1	0	0	1
NC_018732	D1	3025637	0	2	0	2
NC_018732	D1	3088676	0	0	1	1
NC_018732	D1	4500008	0	0	1	1
NC_018732	D1	5920435	1	1	0	2
NC_018732	D1	7411697	0	2	1	3
NC_018732	D1	9454782	1	0	0	1
NC_018732	D1	12232454	4	3	2	9
NC_018732	D1	16938308	2	2	0	4
NC_018732	D1	28874878	1	0	0	1

NC_018732	D1	29577255	3	2	0	5
NC_018732	D1	34510046	6	5	7	18
NC_018732	D1	37925792	2	2	1	5
NC_018732	D1	38627989	0	0	1	1
NC_018732	D1	41131499	1	0	1	2
NC_018732	D1	42346634	1	1	2	4
NC_018732	D1	44944640	2	2	0	4
NC_018732	D1	51720011	1	1	0	2
NC_018732	D1	53734721	2	1	1	4
NC_018732	D1	53734833	0	1	0	1
NC_018732	D1	59464120	2	3	1	6
NC_018732	D1	60173856	0	0	1	1
NC_018732	D1	63310574	0	2	0	2
NC_018732	D1	70433473	1	0	2	3
NC_018732	D1	70485763	1	0	1	2
NC_018732	D1	71982781	0	0	1	1
NC_018732	D1	74686155	1	0	0	1
NC_018732	D1	76871495	2	1	0	3
NC_018732	D1	82086638	4	2	1	7
NC_018732	D1	84874831	4	2	2	8
NC_018732	D1	86230965	0	0	1	1
NC_018732	D1	94144838	6	6	6	18
NC_018732	D1	96603565	0	1	0	1
NC_018732	D1	108715901	0	0	1	1
NC_018732	D1	108968580	0	0	1	1
NC_018732	D1	108968975	1	1	1	3
NC_018732	D1	109552465	1	2	0	3
NC_018732	D1	110348097	0	0	1	1
NC_018732	D1	110448551	1	2	0	3
NC_018732	D1	113457614	0	2	0	2
NC_018732	D1	117069883	1	0	0	1
NC_018732	D1	117070012	0	1	1	2
NC_018732	D1	117070342	6	4	5	15
NC_018732	D1	117084721	1	2	0	3
NC_018732	D1	117546889	1	0	0	1
NC_018733	D2	1922130	3	6	6	15
NC_018733	D2	5434479	0	0	1	1
NC_018733	D2	11006117	0	2	1	3
NC_018733	D2	12595596	5	4	6	15
NC_018733	D2	14815517	1	0	0	1
NC_018733	D2	17084228	0	0	1	1
NC_018733	D2	17084672	3	2	1	6
NC_018733	D2	31998612	0	1	0	1
NC_018733	D2	32724794	0	1	2	3
NC_018733	D2	40076407	0	0	1	1

NC_018733	D2	40076846	1	1	0	2
NC_018733	D2	44308032	0	1	0	1
NC_018733	D2	44490435	0	0	1	1
NC_018733	D2	47013868	1	0	0	1
NC_018733	D2	54315485	0	0	1	1
NC_018733	D2	64430411	0	2	0	2
NC_018733	D2	66588965	0	1	0	1
NC_018733	D2	69077746	1	1	0	2
NC_018733	D2	81000093	3	3	3	9
NC_018733	D2	85309589	1	0	0	1
NC_018733	D2	85735155	0	1	0	1
NC_018734	D3	38056	1	0	0	1
NC_018734	D3	3280955	1	0	0	1
NC_018734	D3	8170704	2	1	0	3
NC_018734	D3	13424950	1	0	0	1
NC_018734	D3	13425087	2	1	0	3
NC_018734	D3	18283759	1	0	0	1
NC_018734	D3	19110019	1	0	0	1
NC_018734	D3	20727107	4	3	1	8
NC_018734	D3	20727779	0	0	1	1
NC_018734	D3	20932216	0	0	2	2
NC_018734	D3	21527671	0	0	1	1
NC_018734	D3	26724053	1	0	0	1
NC_018734	D3	33813677	0	0	1	1
NC_018734	D3	33864506	0	0	1	1
NC_018734	D3	41416368	1	1	0	2
NC_018734	D3	43596760	0	0	1	1
NC_018734	D3	50258483	1	1	1	3
NC_018734	D3	55004170	0	1	0	1
NC_018734	D3	56009888	1	2	3	6
NC_018734	D3	56659432	0	0	1	1
NC_018734	D3	58399775	0	0	1	1
NC_018734	D3	64560362	4	3	3	10
NC_018734	D3	66649986	0	0	1	1
NC_018734	D3	67220746	0	0	1	1
NC_018734	D3	69552699	0	0	1	1
NC_018734	D3	69553669	0	0	1	1
NC_018734	D3	70892634	1	0	0	1
NC_018734	D3	70896943	3	3	1	7
NC_018734	D3	71626677	1	0	1	2
NC_018734	D3	71626705	4	3	4	11
NC_018734	D3	72139658	0	0	1	1
NC_018734	D3	77608060	1	0	0	1
NC_018734	D3	79002969	2	1	0	3
NC_018734	D3	84470684	5	3	3	11

NC_018734	D3	91938287	1	0	0	1
NC_018734	D3	96654458	3	3	2	8
NC_018734	D3	96668941	0	0	1	1
NC_018735	D4	68837	0	1	0	1
NC_018735	D4	77279	0	0	1	1
NC_018735	D4	2891083	0	2	0	2
NC_018735	D4	6886167	1	0	0	1
NC_018735	D4	10921105	1	2	1	4
NC_018735	D4	14464243	3	4	3	10
NC_018735	D4	20749578	0	0	2	2
NC_018735	D4	20749606	3	2	3	8
NC_018735	D4	26228590	3	1	1	5
NC_018735	D4	30619295	1	2	1	4
NC_018735	D4	35058517	1	2	0	3
NC_018735	D4	56518664	3	2	1	6
NC_018735	D4	62779017	4	2	0	6
NC_018735	D4	64421742	0	1	0	1
NC_018735	D4	64430176	0	1	0	1
NC_018735	D4	65594559	2	1	0	3
NC_018735	D4	66602892	0	0	1	1
NC_018735	D4	67395473	0	0	1	1
NC_018735	D4	69536905	0	0	1	1
NC_018735	D4	75206367	1	1	2	4
NC_018735	D4	75719625	0	0	1	1
NC_018735	D4	75720081	4	6	6	16
NC_018735	D4	76548648	5	6	6	17
NC_018735	D4	81211693	1	0	0	1
NC_018735	D4	83528696	1	0	0	1
NC_018735	D4	85158585	0	1	0	1
NC_018735	D4	90423961	0	0	1	1
NC_018735	D4	90952814	1	0	0	1
NC_018735	D4	93281632	1	0	0	1
NC_018735	D4	94404357	0	1	0	1
NC_018735	D4	94408529	0	2	2	4
NC_018735	D4	94409684	2	2	6	10
NC_018736	E1	7054245	0	1	0	1
NC_018736	E1	13053580	0	1	0	1
NC_018736	E1	18765073	0	1	0	1
NC_018736	E1	27459409	0	1	0	1
NC_018736	E1	31022441	0	1	0	1
NC_018736	E1	31242442	1	1	0	2
NC_018736	E1	34550893	0	2	1	3
NC_018736	E1	34635825	0	0	1	1
NC_018736	E1	38355138	0	1	0	1
NC_018736	E1	38805022	0	1	1	2

NC_018736	E1	39851278	0	1	0	1
NC_018736	E1	44063114	1	0	0	1
NC_018736	E1	45368198	3	6	5	14
NC_018737	E2	948647	0	2	2	4
NC_018737	E2	5650621	2	3	3	8
NC_018737	E2	5779387	1	0	0	1
NC_018737	E2	9851136	0	2	3	5
NC_018737	E2	11098212	1	1	0	2
NC_018737	E2	15747326	0	1	0	1
NC_018737	E2	21049723	2	1	0	3
NC_018737	E2	23620399	5	5	5	15
NC_018737	E2	23620850	0	1	1	2
NC_018737	E2	25988565	0	0	1	1
NC_018737	E2	29101585	6	6	7	19
NC_018737	E2	29101989	1	0	0	1
NC_018737	E2	31833384	1	1	1	3
NC_018737	E2	43156547	0	0	1	1
NC_018737	E2	49129125	0	1	0	1
NC_018737	E2	49185667	0	0	1	1
NC_018737	E2	49185925	0	0	1	1
NC_018737	E2	49407286	0	0	1	1
NC_018737	E2	51149544	1	2	2	5
NC_018737	E2	51190684	1	1	1	3
NC_018737	E2	51191294	0	0	1	1
NC_018737	E2	54244709	1	0	0	1
NC_018737	E2	54522593	1	0	0	1
NC_018737	E2	60173567	4	4	4	12
NC_018737	E2	63909683	0	2	0	2
NC_018737	E2	64322551	0	0	1	1
NC_018738	E3	144548	5	4	6	15
NC_018738	E3	5967351	4	3	3	10
NC_018738	E3	9267181	0	1	0	1
NC_018738	E3	13735545	0	1	1	2
NC_018738	E3	14901325	4	3	3	10
NC_018738	E3	19845331	0	0	1	1
NC_018738	E3	20648883	0	1	1	2
NC_018738	E3	22217307	1	1	0	2
NC_018738	E3	23632505	0	0	1	1
NC_018738	E3	27492107	1	0	0	1
NC_018738	E3	34448052	0	2	0	2
NC_018738	E3	41617012	1	0	0	1
NC_018738	E3	43697041	2	2	0	4
NC_018739	F1	6513161	2	3	0	5
NC_018739	F1	6513397	1	0	0	1
NC_018739	F1	10233702	4	3	3	10

NC_018739	F1	11537322	0	0	1	1
NC_018739	F1	11537448	1	1	0	2
NC_018739	F1	11542267	1	0	0	1
NC_018739	F1	11542535	1	2	2	5
NC_018739	F1	11542643	4	4	5	13
NC_018739	F1	13581163	0	1	0	1
NC_018739	F1	15650031	0	1	0	1
NC_018739	F1	15793664	0	0	1	1
NC_018739	F1	18740066	6	5	6	17
NC_018739	F1	18740519	0	0	1	1
NC_018739	F1	23153256	2	0	1	3
NC_018739	F1	25061097	0	0	1	1
NC_018739	F1	25497226	0	0	1	1
NC_018739	F1	25497659	0	0	1	1
NC_018739	F1	26576310	0	0	1	1
NC_018739	F1	27652555	0	1	0	1
NC_018739	F1	34209671	0	1	0	1
NC_018739	F1	34758843	1	0	2	3
NC_018739	F1	39462021	0	0	1	1
NC_018739	NC_018739	44569759	1	0	1	2
NC_018739	F1	44636796	0	0	1	1
NC_018739	F1	44637016	6	6	6	18
NC_018739	F1	44965636	1	0	0	1
NC_018739	F1	50137420	0	0	1	1
NC_018739	F1	50624373	3	2	5	10
NC_018739	F1	51873005	0	1	0	1
NC_018739	F1	53355424	4	3	4	11
NC_018739	F1	54567121	4	2	1	7
NC_018739	F1	54591133	1	0	0	1
NC_018739	F1	56676514	1	0	1	2
NC_018739	F1	61873554	0	1	0	1
NC_018739	F1	62043991	0	0	1	1
NC_018740	F2	5512050	0	0	1	1
NC_018740	F2	8206984	1	0	0	1
NC_018740	F2	19101086	4	2	5	11
NC_018740	F2	20557764	0	0	1	1
NC_018740	F2	25915820	1	0	0	1
NC_018740	F2	29985010	0	0	1	1
NC_018740	F2	30387404	5	3	2	10
NC_018740	F2	32802780	1	1	0	2
NC_018740	F2	32926553	2	3	3	8
NC_018740	F2	44672763	0	0	1	1
NC_018740	F2	57246774	0	1	0	1
NC_018740	F2	63221837	0	0	1	1
NC_018740	F2	63222033	3	2	3	8

NC_018740	F2	67208674	4	3	0	7
NC_018740	F2	79026042	1	0	0	1
NC_018740	F2	84321425	2	0	0	2
NC_018740	F2	85487888	1	0	1	2
NC_018740	F2	85487943	5	3	5	13
NC_018740	F2	85604433	0	0	1	1
NC_018741	X	75504	3	5	5	13
NC_018741	X	1254459	2	3	3	8
NC_018741	X	1497462	0	0	1	1
NC_018741	X	1789828	0	1	0	1
NC_018741	X	6656738	0	0	1	1
NC_018741	X	7596947	3	1	0	4
NC_018741	X	8831199	6	6	6	18
NC_018741	X	8831316	0	0	1	1
NC_018741	X	8839499	2	3	3	8
NC_018741	X	8839619	2	0	2	4
NC_018741	X	19037404	1	0	0	1
NC_018741	X	33735067	1	0	0	1
NC_018741	X	34680718	0	1	0	1
NC_018741	X	34733171	0	1	0	1
NC_018741	X	45785579	1	1	1	3
NC_018741	X	45785698	3	3	4	10
NC_018741	X	47503465	0	0	1	1
NC_018741	X	52608333	1	2	1	4
NC_018741	X	52608515	4	3	3	10
NC_018741	X	56013098	1	1	0	2
NC_018741	X	56059326	0	1	0	1
NC_018741	X	56061010	0	1	0	1
NC_018741	X	63738209	0	0	1	1
NC_018741	X	76040125	0	2	0	2
NC_018741	X	80017221	1	1	0	2
NC_018741	X	84736914	0	1	0	1
NC_018741	X	84744593	4	2	1	7
NC_018741	X	91590709	0	3	0	3
NC_018741	X	95157426	1	0	0	1
NC_018741	X	95727165	0	2	2	4
NC_018741	X	98027273	5	3	2	10
NC_018741	X	111416458	3	3	0	6
NC_018741	X	121948744	1	1	0	2
NC_018741	X	122403862	5	6	6	17
NC_018741	X	124161719	1	1	0	2
NC_018741	X	126845244	0	0	1	1
NC_018741	X	126845601	5	2	1	8
NC_018741	X	127008044	1	0	0	1
NC_018741	X	127008046	1	0	0	1
