# Supplementary material for: Endogenous feline leukemia virus long terminal repeat integration site diversity is highly variable in related and unrelated domestic cats
Source: Retrovirology. 2024 Feb 12;21:3. doi: 10.1186/s12977-024-00635-0 (PMC10863107; doi:10.1186/s12977-024-00635-0)
Supplement: Supplementary file 8 — Additional file 8: Table S2. 960 protein-coding genes are associated with all 80 integration sites found in at least ten individuals. [file 12977_2024_635_MOESM8_ESM.pdf]

Accession No.	Chromosome	Integration site	Number of cats	Gene
NC_018723	A1	37559850	14	TDRD3
NC_018723	A1	87434194	19	MTUS2
NC_018723	A1	87661908	18	MTUS2
NC_018723	A1	91986082	18	ubiquitin like 3
NC_018723	A1	125666538	11	CA1H5orf46
				CCNO
				CDC20B
				DDX4
				DHX29
				DPYSL3
				GPX8
				GZMA
				IL31RA
				IL6ST
				JAKMIP2
				MARCOL
				MCIDAS
				MTREX
				PLPP1
				PPP2R2B
				SCGB3A2
				SLC38A9
				SPINK1
				SPINK14
				SPINK5
				SPINK6
				STK32A
NC_018723	A1	154602427	10	MEF2C
				CETN3
				MBLAC2
				POLR3G
				LYSMD3
				ADGRV1
				ARRDC3
NC_018723	A1	241024241	16	AHRR
				BRD9
				CA1H5orf38
				CCDC127
				CEP72
				EXOC3
				IRX2
				IRX4
				LPCAT1
				LRRC14B
				MRPL36
				NDUFS6
				NKD2
				PDCD6
				SDHA
				SLC12A7
				SLC6A3
				SLC9A3
				TERT
				TPPP
				TRIP13
NC_018724	A2	7199456	19	None
NC_018724	A2	48890477	10	GRM7
NC_018724	A2	56416655	14	ACAD9
				CNBP
				COPG1
				EFCC1
				FBLN2
				GP9
				HDAC11
				HMCES
				IQSEC1
				ISY1
				KIAA1257
				NUP210
				RAB43
				RAB7A
				WNT7A
NC_018724	A2	70794560	15	EGFR
				LANCL2
				POM121L12
				SEC61G
				VOPP1
				VSTM2A
NC_018724	A2	123657107	11	ADCYAP1R1
				AQP1
				CCDC129
				CRHR2
				FKBP14
				GARS
				GGCT
				GHRHR
				MINDY4
				MTURN
				NEUROD6
				NOD1
				PLEKHA8
				PPP1R17
				PRR15
				SCRN1
				WIPF3
				ZNRF2
NC_018724	A2	143119473	14	ASB15
				GPR37
				HYAL4
				IQUB
				LMOD2
				NDUFA5
				POT1
				SLC13A1
				SPAM1
				TAS2R16
				TMEM229A
				WASL
				MYT1
				PCMTD2
				NPBWR2

NC_018725	A3	97444	15	LKAAERA1<br>ABHD16B<br>CHRNA4<br>DNAJC5<br>EEF1A2<br>FNDC11<br>GMEB2<br>HELZ2<br>KCNQ2<br>LIME1<br>OPRL1<br>PPDPF<br>PRPF6<br>PTK6<br>RGS19<br>RTEL1<br>SAMD10<br>SLC2A4RG<br>SOX18<br>SRMS<br>STMN3<br>TCEA2<br>TNFRSF6B<br>TPD52L2<br>UCKL1<br>ZBTB46<br>ZGPAT<br>ZNFS12B
NC_018725	A3	3320238	20	None
NC_018725	A3	38977257	10	SPTLC3<br>ISM1<br>TASP1
NC_018725	A3	77888994	11	None
NC_018725	A3	77917349	18	CCDC85A<br>EFEMP1<br>FANCL<br>VRK2
NC_018725	A3	102855515	12	ATOH8<br>CA3H2orf68<br>CAPG<br>CD8A<br>ELMOD3<br>GGCX<br>IMMT<br>KCMF1<br>KDM3A<br>MAT2A<br>MRPL35<br>POLR1A<br>PTCD3<br>REEP1<br>RETSAT<br>RMND5A<br>RNF181<br>SFTPB<br>SH2D6<br>ST3GAL5<br>TCF7L1<br>TGOLN2<br>TMEM150A<br>TMSB10<br>TRABD2A<br>TRNAK-CUU<br>USP39<br>VAMP5<br>VAMP8
NC_018725	A3	133841631	11	TRIB2
NC_018725	A3	142200005	11	ACP1<br>ALKAL2<br>FAM110C<br>MYT1L<br>PXDN<br>SH3YL1<br>SNTG2<br>TMEM18<br>TPO
NC_018726	B1	67270589	19	FSTL5<br>NAF1<br>NPY1R<br>NPY5R<br>TKTL2<br>TMA16
NC_018726	B1	124950589	10	MTTP<br>TRMT10A<br>CB1H4orf17<br>ADH5<br>METAP1<br>EIF4E<br>TSPAN5
NC_018726	B1	136552325	15	AFF1<br>CB1H4orf36<br>HSD17B11<br>HSD17B13<br>KLHL8<br>MAPK10<br>NUDT9<br>PRPN13<br>SLC10A6
NC_018726	B1	156352163	11	ADGRL3<br>TECRL
NC_018726	B1	201827018	18	CYTL1<br>DRD5<br>EVC<br>EVC2<br>LYAR<br>MSX1<br>NSG1<br>OTOP1<br>SLC2A9<br>STK32B<br>STX18<br>TMEM128

				TRNAK-CUU
				WDR1
				ZBTB49
				ZNF518B
NC_018726	B1	207201855	17	ADD1
				ATP5ME
				CB1H4orf48
				CFAP99
				CPLX1
				CTBP1
				DGKQ
				FAM193A
				FGFR3
				FGFRL1
				GAK
				HAUS3
				IDUA
				LETM1
				MAEA
				MXD4
				MYL5
				NAT8L
				NELFA
				NKX1-01
				NSD2
				PCGF3
				PDE68
				PIGG
				POLN
				RNF212
				RNF4
				SH3BP2
				SLBP
				SLC49A3
				SPON2
				TACC3
				TMEM129
				TMEM175
				TNIP2
				UVSSA
				ZFYBE28
NC_018727	B2	28112801	11	FOXQ1
				EXOC2
				HUS1B
NC_018727	B2	32150838	19	ABCF1
				ABHD16A
				AGER
				AGPAT1
				AIF1
				APOM
				ATAT1
				ATF6B
				ATP6V1G2
				BAG6
				C2
				CB2H6orf136
				CB2H6orf47
				CCHCR1
				CCHCR1
				CDSN
				CFB
				CLIC1
				CSNK2B
				DDAH2
				DDR1
				DDX39B
				DHX16
				DPCR1
				DXO
				EGFL8
				EHMT2
				FKBPL
				FLOT1
				GNL1
				GPANK1
				GPSM3
				GTF2H4
				IER3
				LSM2
				LST1
				LY6G5B
				LY6G5C
				LY6G6C
				MCCD1
				MDC1
				MPIG6B
				MRPS18B
				MSH5
				MUC21
				NCR3
				NELFE
				NEU1
				NFKBIL1
				NOTCH4
				NRM
				PBX2
				POU5F1
				PPP1R10
				PPP1R18
				PPT2
				PRR3
				PRRC2A
				PRRT1
				PSORS1C2
				RNF5
				SAPCD1
				SFTA2
				SKIV2L
				SLC44A4
				STK19
				TCF19
				TNF
				TUBB

				VARS
				VARS2
				VWA7
				ZBTB12
NC_018727	B2	55690560	20	HCRTR2
				GFRAL
				HMGCLL1
				BMP5
NC_018727	B2	65730248	19	LMBRD1
				COL19A1
NC_018727	B2	140538176	12	ESR1
				FBXO5
				MTRF1L
				MYCT1
				RGS17
				SYNE1
				VIP
NC_018727	B2	146684232	20	ACAT2
				AGPAT4
				DYNLT1
				EZR
				FNDC1
				IGF2R
				MAP3K4
				MAS1
				MRPL18
				PLG
				PNLDC1
				RSPH3
				SLC22A1
				SLC22A3
				SOD2
				SYTL3
				TAGAP
				TCP1
				TMEM181
				WTAP
NC_018727	B2	150861037	16	ACSBG1
				ADAMTS7
				CB2H6orf118
				CHRNA3
				CHRNA5
				CHRNB4
				CTSH
				DNAJA4
				HYKK
				IDH3A
				IREB2
				MORF4L1
				PSMA4
				QKI
				RASGRF1
				SH2D7
				TBC1D2B
				TRNAK-CUU
				WDR61
NC_018728	B3	2130434	11	ARNT2
				ABHD17C
				CEMIP
				MESD
				TLNRD1
				CFAP161
				IL16
				STARD5
				TMC3
NC_018728	B3	25546595	10	ATP10A
				GABRA5
				GABRB3
				GABRG3
				TRNAE-UUC
NC_018728	B3	74694912	18	DCKDHB
				ELOVL4
				HMGN3
				LCA5
				PHIP
				SH3BGRL2
				TTK
NC_018728	B3	79470596	19	CEP162
				NT5E
				SNX14
				SYNCRIP
				TBX18
NC_018728	B3	136800478	12	ATXN3
				CALM1
				CATSPERB
				CCDC88C
				CPSF2
				DGLUCY
				FBLN5
				GPR68
				KCNK13
				NDUFB1
				NRDE2
				PPP4R3A
				PSMC1
				RPS6KA5
				SLC24A4
				TC2N
				TRIP11
				TTC7B
NC_018729	B4	14689014	10	FAM171A1
				ITGA8
				MINDY3
NC_018729	B4	24617796	12	ABI1
				ACBD5
				ANKRD26
				APBB1IP
				GAD2
				MASTL
				MYO3A
				PDSS1
				PTCHD3

				RAB18
				YME1L1
NC_018729	B4	29973213	13	ARHGAP12
				CCDC7
				EPC1
				KIF5B
				NRP1
NC_018729	B4	64351845	18	CCDC91
				ERGIC2
				FAR2
				OVCH1
				TMTC1
NC_018729	B4	79102334	11	FMNL3
				TMBIM6
				NCKAP5L
				BCDIN3D
				FAIM2
				AQP2
				AQP5
				AQP6
				RACGAP1
				ASIC1
				SMARCD1
				GPD1
				TRNAK-CUU
				CERS5
				LIMA1
				FAM186A
				LARP4
				DIP2B
				ATF1
NC_018730	C1	46392926	10	TRNAK-CUU
				PLPP3
				PRKAA2
				FYB2
				C8A
				C8B
NC_018730	C1	58764761	12	ANKRD13C
				CTH
				LRRC40
				LRRC7
				PTGER3
				SRSF11
				ZRANB2
NC_018730	C1	68452850	10	ADGRL2
NC_018730	C1	219236324	12	COPS8
				COL6A3
				MLPH
				PRLH
				RAB17
				LRRFIP1
				RBM44
				RAMP1
NC_018731	C2	4816127	15	ABI3BP
				ADGRG7
				CMSS1
				COL8A1
				FILIP1L
				NIT2
				TBC1D23
				TFG
				TMEM45A
				TOMM70
NC_018731	C2	131951595	15	ACAD11
				ACPP
				BFSP2
				CDV3
				DNAJC13
				NPHP3
				RAB6B
				SLCO2A1
				SRPRB
				TMEM108
				TOPBP1
				tRNAC-GCA
NC_018731	C2	135744902	11	ANKRD28
				BTD
				CAPN7
				COLQ
				DAZL
				DPH3
				EAF1
				GALNT15
				HACL1
				METTL6
				OXNAD1
				PLCL2
				RFTN1
				SH3BP5
NC_018731	C2	143414052	11	THRB
NC_018731	C2	160924755	18	ABHD5
				ANO10
				TCAIM
				TOPAZ1
				ZNF35
				ZNF445
NC_018732	D1	34510046	18	ARHGAP42
				BET1L
				MUC2
				MUC5B
				NLRP6
				ODF3
				PGGHG
				PGR
				RIC8A
				SCGB1C1
				SIRT3
				TOLLIP
NC_018732	D1	94144838	18	None
				ANO9
				AP2A2
				B4GALNT4

NC_018732	D1	117070342	15	BRSK2
				CDHR5
				CEND1
				CHID1
				CRACR2B
				CTSD
				DEAF1
				DRD4
				DUSP8
				EPS8L2
				GATD1
				HRAS
				IFITM10
				IFITM5
				IRF7
				LMNTD2
				LRRC56
				LSP1
				MOB2
				MRPL23
				PANO1
				PHRF1
				PIDD1
				PKP3
				PNPLA2
				POLR2L
				PTDSS2
				RASSF7
				RNH1
				RPLP2
				SCT
				SIGIRR
				SLC25A22
				SYT8
				TALDO1
				TMEM80
				TNNI2
				TNNT3
				TSPAN4
NC_018733	D2	1922130	15	ZWINT
NC_018733	D2	12595596	15	ACTN2
				B3GALNT2
				EDARADD
				ERO1B
				GNG4
				GPR137B
				HEATR1
				LGALS8
				LYST
				MTR
				NID1
				RYR2
NC_018734	D3	64560362	10	PIK3C3
NC_018734	D3	71626705	11	ACAA2
				CD3H18orf32
				CFAP53
				CXXC1
				DYM
				ELAC1
				LIPG
				MAPK4
				MBD1
				ME2
				MRO
				MYO5B
				RPL17
				SKA1
				SMAD4
				SMAD7
NC_018734	D3	84470684	11	CDH7
				SERPINB10
				SERPINB11
				SERPINB12
				SERPINB13
				SERPINB2
				SERPINB3
				SERPINB5
				SERPINB7
NC_018735	D4	14464243	10	ABHD17B
				ALDH1A1
				ANXA1
				CD4H9orf85
				GDA
				TMC1
				TMEM2
				TRPM3
				ZFAND5
NC_018735	D4	75720081	16	AKNA
				ALAD
				AMBP
				ATP6V1G1
				BSPRY
				CD4H9orf43
				CDC26
				COL27A1
				FKBP15
				HDHD3
				INIP
				KIAA1958
				KIF12
				ORM2
				POLE3
				PRPF4
				RGS3
				RNF183
				SLC31A1
				SLC31A2
				SLC46A2
				SNX30
				TEX48
				TMEM268
				WDR31
				WHRN

				ZFP37
				ZNF618
				ZNF883
NC_018735	D4	76548648	17	AKNA
				AMBP
				ATP6V1G1
				COL27A1
				KIF12
				ORM2
				RGS3
				TEX48
				TMEM268
				TNC
				TNFSF15
				TNFSF8
				WHRN
				ZNF618
NC_018735	D4	94409684	10	SARDH
				DBH
				FAM163B
				ADAMTS13
				ADAMTSL2
				AGPAT2
				BLGI
				BLGIII
				BRD3
				CACFD1
				CAMSAP1
				CARD9
				CCDC187
				DBH
				DNLZ
				EGFL7
				FAM163B
				FAM69B
				GLT6D1
				GPSM1
				INPP5E
				KCNT1
				LCN9
				LHX3
				MED22
				MYMK
				NACC2
				NOTCH1
				PMPCA
				QSOX2
				REXO4
				RPL7A
				RXRA
				SARDH
				SDCCAG3
				SEC16A
				SLC2A6
				SNAPC4
				SOHLH1
				STKLD1
				SURF2
				SURF4
				SURF6
				TMEM250
				NACC2
NC_018736	E1	45368198	14	ACBD4
				ADAM11
				ARHGAP27
				C1QL1
				CCDC103
				CCDC43
				CRHR1
				DBF4B
				DCAKD
				EFTUD2
				FAM171A2
				FMNL1
				FZD2
				GFAP
				GJC1
				GOSR2
				GPATCH8
				GRN
				HEXIM1
				HIGD1B
				ITGA2B
				KANSL1
				KIF18B
				LYZL6
				MAP3K14
				MAPT
				MEIOC
				NMT1
				NSF
				PLCD3
				PLEKHM1
				RPRML
				SLC25A39
				SPATA32
				SPPL2C
				WNT3
				WNT9B
NC_018737	E2	23620399	15	CCNE1
				CE2H19orf12
				PLEKHF1
				POP4
				URI1
				VSTM2B
NC_018737	E2	29101585	19	ABCC11
				ABCC12
				CBLN1
				CE2H16orf78
				LONP2
				N4BP1
				SIAH1
				ZNF423

NC_018737	E2	60173567	12	TLDC1
				COTL1
				CRISPLD2
				ZDHHC7
				KIAA0513
				FAM92B
				GINS2
				CE2H16orf74
NC_018738	E3	144548	15	ADAP1
				CE3H7orf50
				DNAAF5
				FAM20C
				GET4
				GPER1
				GPR146
				PDGFA
				PRKAR1B
				SUN1
				ZFAND2A
NC_018738	E3	5967351	10	CCZ1
				LMTK2
				BHLHA15
				TECPR1
				BRI3
				BAIAP2L1
				NPTX2
				TMEM130
				TRRAP
				SMURF1
NC_018738	E3	14901325	10	CALN1
NC_018739	F1	10233702	10	BECN2
				EXO1
				WDR64
				OPN3
				CHML
				KMO
				FH
				RGS7
NC_018739	F1	11542643	13	FMN2
				POGK
				TADA1
				ILDR2
				MAEL
				GPA33
				DUSP27
NC_018739	F1	18740066	17	CACYBP
				CENPL
				DARS2
				GPR52
				KIAA0040
				KLHL20
				MRPS14
				RABGAP1L
				RC3H1
				SERPINC1
				TNN
				ZBTB37
NC_018739	F1	44637016	18	ADORA1
				ATP284
				BTG2
				CHI3L1
				CHIT1
				CNTN2
				DSTYK
				ETNK2
				FMOD
				GOLT1A
				KISS1
				LAX1
				LRRN2
				MDM4
				MYBPH
				MYOG
				NFASC
				OPTC
				PIK3C23
				PLEKHA6
				PPFIA4
				PPP1R15B
				PRELP
				RBBP5
				REN
				SNRPE
				SOX13
				TMCC2
				TMEM81
				TRNAN-GUU
				ZBED6
				ZC3H11A
NC_018739	F1	50624373	10	CDC73
				GLRX2
				TROVE2
				UCHL5
				RGS2
				RGS13
				RGS1
				RGS18
NC_018739	F1	53355424	11	None
NC_018740	F2	19101086	11	TRNAR-ACG
				TRIM55
				CRH
				ADHFE1
				CF2H8orf46
				MYBL1
				VCPIP1
				SGK3
				TCF24
				PPP1R42
				COPS5
				CSPP1
NC_018740	F2	30387404	10	STMN2
				HEY1

				MRPS28
NC_018740	F2	85487943	13	ADCK5
				ARHGAP39
				BOP1
				CCDC166
				CF2H8orf33
				CF2H8orf82
				COMMD5
				CPSF1
				CYHR1
				DGAT1
				EEF1D
				EPPK1
				EXOSC4
				FAM83H
				FBXL6
				FOXH1
				GPAA1
				GPT
				GRINA
				GSDMD
				HGH1
				HSF1
				IQANK1
				KIFC2
				LRRC14
				LRRC24
				MAF1
				MAFA
				MAPK15
				MFSD3
				MROH1
				MROH6
				NAPRT
				NRBP2
				OPLAH
				PARP10
				PLEC
				PPP1R16A
				PUF60
				PYCR3
				RECQL4
				RPL8
				SCRIB
				SCRT1
				SCX
				SHARPIN
				SLC39A4
				SLC52A2
				SMPD5
				SPATC1
				TIGD5
				TMEM249
				TONSL
				TSTA3
				VPS28
				WDR97
				ZC3H3
				ZNF16
				ZNF250
				ZNF34
				ZNF517
				ZNF623
				ZNF7
