# Supplementary material for: Endogenous feline leukemia virus long terminal repeat integration site diversity is highly variable in related and unrelated domestic cats
Source: Retrovirology. 2024 Feb 12;21:3. doi: 10.1186/s12977-024-00635-0 (PMC10863107; doi:10.1186/s12977-024-00635-0)
Supplement: Supplementary file 11 — Additional file 11: Table S5. Significantly differentially expressed genes in FeLV-negative and FeLV-positive fibroblasts. [file 12977_2024_635_MOESM11_ESM.docx]

**Table S5.** Significantly differentially expressed genes in FeLV-negative and FeLV-positive fibroblasts.

| **Genome** | **Gene name** | **logFC** | **FDR** | **Protein** | **blastn homology** | **distance to nearest LTR integration site** |
| --- | --- | --- | --- | --- | --- | --- |
| FeLV | FeLVgp1 | 10.72 | 1.18E-42 | Gag-Pro-Pol precursor polyprotein gPr80 | na | na |
| FeLV | FeLVgp2 | 9.78 | 2.72E-30 | Env polyprotein | na | na |
| *Felis catus* | LOC111561459 | 6.12 | 1.01E-18 | uncharacterized protein LOC111561459 | gag-pro-pol (93.1%) | 519 |
| *Felis catus* | LOC105260391 | 6.41 | 2.42E-13 | uncharacterized protein LOC105260391 | gag-pro-pol (90.3%) | 35,225 |
| *Felis catus* | LOC109496917 | 2.75 | 1.40E-03 | uncharacterized protein LOC109496917 | env (96.1%) | 5,776 |
